# A Double-Blind, Randomized Controlled, Acute Feeding Equivalence Trial of Small, Catalytic Doses of Fructose and Allulose on Postprandial Blood Glucose Metabolism in Healthy Participants: The Fructose and Allulose Catalytic Effects (FACE) Trial

**DOI:** 10.3390/nu10060750

**Published:** 2018-06-09

**Authors:** Catherine R. Braunstein, Jarvis C. Noronha, Andrea J. Glenn, Effie Viguiliouk, Rebecca Noseworthy, Tauseef A. Khan, Fei Au-Yeung, Sonia Blanco Mejia, Thomas M.S. Wolever, Robert G. Josse, Cyril W.C. Kendall, John L. Sievenpiper

**Affiliations:** 1 Toronto 3D Knowledge Synthesis and Clinical Trials Unit, Risk Factor Modification Centre, Toronto, ON M5C 2T2, Canada; catherine.braunstein@mail.utoronto.ca (C.R.B.); jarvis.noronha@mail.utoronto.ca (J.C.N.); andrea.glenn@alum.utoronto.ca (A.J.G.); effie.viguiliouk@mail.utoronto.ca (E.V.); tauseef.khan@utoronto.ca (T.A.K.); rodney.auyeung@mail.utoronto.ca (F.A.-Y.); sonia.blancomejia@mail.utoronto.ca (S.B.M.); thomas.wolever@utoronto.ca (T.M.S.W.); josserg@smh.ca (R.G.J.); cyril.kendall@utoronto.ca (C.W.C.K.); 2 Department of Nutritional Sciences, Faculty of Medicine, University of Toronto, Toronto, ON M5S 3E2, Canada; rebecca.noseworthy@utoronto.ca; 3 Department of Medicine, Faculty of Medicine, University of Toronto, Toronto, ON M5S 3E2, Canada; 4 Li Ka Shing Knowledge Institute, St. Michael’s Hospital, Toronto, ON M5B 1T8, Canada; 5 Division of Endocrinology and Metabolism, St. Michael’s Hospital, Toronto, ON M5C 2T2, Canada; 6 College of Pharmacy and Nutrition, University of Saskatchewan, Saskatoon, SK S7N 5C9, Canada

**Keywords:** D-allulose, D-psicose, D-fructose, catalytic effects, postprandial blood glucose regulation

## Abstract

Recent literature suggests that catalytic doses (≤10 g/meal or 36 g/day) of D-fructose and D-allulose may reduce postprandial blood glucose responses to carbohydrate loads in people with and without type 2 diabetes by inducing glycogen synthesis. To assess the effect of small single doses of fructose and allulose on postprandial blood glucose regulation in response to a 75 g-oral glucose tolerance test (75 g-OGTT) in healthy individuals, we conducted an acute randomized, crossover, equivalence trial in healthy adults. Each participant randomly received six treatments, separated by a minimum one-week washout. Treatments consisted of a 75 g-OGTT with the addition of fructose or allulose at 0 g (control), 5 g or 10 g. A standard 75 g-OGTT protocol was followed with blood samples at −30, 0, 30, 60, 90, 120 min. The primary outcome was the difference in plasma glucose incremental area under the curve (iAUC). A total of 27 participants underwent randomization with data available from 25 participants. Small doses of fructose or allulose did not show a significant effect on plasma glucose iAUC or other secondary markers of postprandial blood glucose regulation in response to a 75 g-OGTT in healthy individuals. These results were limited by the low power to detect a significant difference, owing to greater than expected intra-individual coefficient of variation (CV) in plasma glucose iAUC. Overall, we failed to confirm the catalytic effects of small doses of fructose and allulose in healthy individuals. Future trials may consider recruiting larger sample sizes of healthy individuals. Trial registration: clinicaltrials.gov identifier, NCT02459834.

## 1. Introduction

International health organizations have called for a reduction in added or free sugar intakes [1,2,3]. This call has created a need for ‘healthy’ alternatives to replace sugars.

Although the fructose moiety of sugars has been implicated for having a role in weight gain and increased diabetes risk [4,5,6,7], there is emerging literature that suggests it may be bimodal in its responses. High doses of fructose have an adverse effect on body weight, fasting blood glucose, and insulin sensitivity among other cardiometabolic risk factors [8,9,10,11,12,13,14]. However, fructose at lower doses in energy matched comparisons with other non-fructose containing carbohydrates has shown improvements in glycemic control [13,14,15,16,17,18]. This apparent benefit has been explained by a ‘catalytic’ effect of fructose on glucose metabolism. Low doses of fructose, such as levels obtainable from fruit (≤10 g/meal) increase glucokinase activity, manifesting in an increase in glycogen synthesis as shown by 13C nuclear magnetic resonance (NMR) under euglycemic conditions [19] and decreasing hepatic glucose output under hyperglycemic clamp conditions [20]. The result is to decrease postprandial blood glucose responses to oral glucose and high glycemic index (GI) carbohydrate meals [21,22,23].

There is an interest in identifying sweeteners that share this ‘catalytic’ mechanism without the adverse effects resulting from excess calories. The C-3 epimer of fructose, allulose (psicose), may have the advantage of sharing this ‘catalytic’ effect without the calories (4 kcal/g of fructose). Allulose is an approved (US FDA, GRAS Notice 693) low-calorie sugar substitute providing ≤0.2 kcal/g compared to 4 kcal/g for sugars [24] that shares many of the functional and sensory properties of sucrose. There is evidence that it can also increase glucokinase activity through the same ‘catalytic’ mechanism resulting in increases in glycogen synthesis that relate to a decrease in the postprandial blood glucose response to high glycemic index carbohydrates [19,20]. 

However, uncertainties do remain. It is unclear whether the catalytic effects of fructose and allulose are equivalent. The minimum dose at which an effect is producible is also unclear. The objective of the present trial was to assess and compare the effects of small, ‘catalytic doses’ (5 g, 10 g) of fructose and allulose on postprandial blood glucose regulation in response to a 75 g-oral glucose tolerance test (75 g-OGTT) in healthy normal-weight individuals without diabetes.

## 2. Materials and Methods

### 2.1. Participants

We included healthy male and female (non-pregnant) volunteers between 18 and 75 years of age with a body mass index (BMI) between 18.5 and 30 kg/m^2^, who were nonsmokers and not using alcohol or drugs heavily. We excluded those with pre-diabetes or diabetes (HbA1c ≥ 6%, FBG ≥ 6.1 mmol/L [25]), any major disease or psychiatric illness; and those who regularly used medications (with the exception of birth control and “as needed” medications, i.e., vitamin/mineral supplements, probiotics, and non-prescription pain medications). Eligible participants provided informed consent. The study protocol was approved by St. Michael’s Research Ethics Review Board, St. Michael’s Hospital, Toronto, Canada (REB# 15-238) and The Mount Sinai Research Ethics Review Board, Mount Sinai Hospital, Toronto, Canada (REB# 16-0229-E) and was registered on ClinicalTrials.gov (NCT02459834).

### 2.2. Trial Design

This trial followed a double-blind, multiple-crossover, randomized, acute feeding equivalence design to assess the effect of small, catalytic doses of fructose and allulose at 0 g, 5 g, and 10 g on postprandial blood glucose regulation in response to a 75 g-OGTT. Randomization of the sequence of the six treatments was done for each participant using a random sequence generator [26]. The study statistician, who was blinded to the identity of the participants and did not have contact with the participants or their data, performed the randomization. Participants, study staff, investigators, and outcome assessors were blinded to the identity of the treatments. There were two levels of allocation concealment. The manufacturer of the treatments (Tate & Lyle Ingredients Americas LLC, Hoffman Estates, IL, USA) provided unique codes for each of the six treatments. The statistician, who was blinded to the identity of these codes, ensured an increased level of blinding by labelling the packaging of the six treatments so that they were only distinguishable by the participant number and their corresponding visit number based on the randomization. The two sets of blinding codes for each participant were not broken until all participants had completed the study and all analyses were completed. 

### 2.3. Treatments

The six treatment drinks were manufactured and provided by Tate & Lyle Ingredients Americas LLC (Hoffman Estates, IL, USA). Treatments consisted of fructose or allulose each at 0 g (control), 5 g, and 10 g added to a 75 g glucose solution dissolved in 500 mL of water [27]. All drinks were designed to appear similar in taste (berry flavour), texture, and appearance (clear/colourless) so that neither the participants nor the study staff could detect the identity of the drinks.

### 2.4. Protocol for Blood Sample Collection

The protocol followed the World Health Organization (WHO) guidelines for the administration of a 75 g-OGTT [28]. Participants came to our outpatient clinic at the Clinical Nutrition and Risk Factor Modification Centre, St. Michael’s Hospital, Toronto, Canada on six separate mornings after a 10 h to 12 h overnight fast with visits scheduled at least one week apart to ensure adequate washout. Participants were asked to maintain their usual diet and physical activity, with instructions to consume at least 150 g of carbohydrates in the 3 days leading up to each visit, and to refrain from excess alcohol consumption (>3 drinks/day) the evening before the visit. A Clinical Assessment Questionnaire was administered by study staff at the beginning of each visit to assess these protocol elements. Side effects were self-reported by the study participants and recorded in daily charts by study staff. The information collected included the frequency and severity of the occurance, the date, and the test drink consumed. Participants who did not follow these pre-visit requirements were asked to repeat the study visit on another day. If a participant followed the pre-visit requirements, then a registered intravenous nurse inserted a catheter into a forearm vein that was secured by tape and kept patent by saline. Heating blankets were used to ensure adequate blood flow over multiple venous blood draws. Two fasting blood samples were taken: one at −30-min and one at 0-min. One of the six treatment drinks was then administered in random order with instructions to consume it at a constant rate over 5 min. Additional samples were taken at 30-min, 60-min, 90-min, and 120-min. Participants received a small monetary compensation and reimbursement of transit fare for their participation.

### 2.5. Outcome Measures

The pre-specified primary outcome measure was the incremental area under the curve (iAUC) for plasma glucose. Pre-specified secondary outcome measures included plasma insulin iAUC, plasma glucose and insulin absolute maximum concentrations (C_max_), time of maximum concentrations (T_max_), and mean incremental concentrations; the Matsuda whole body insulin sensitivity index (Matsuda ISI_OGTT_); and the early insulin secretion index (∆PI_30-0_/∆PG_30-0_). Exploratory outcome measures which were not pre-specified included plasma glucose and insulin total AUC, incremental C_max_, and mean absolute concentrations; and the insulin secretion-sensitivity index-2 (ISSI-2).

### 2.6. Plasma Glucose and Insulin Analyses

Blood samples for glucose and insulin were collected in fluoride oxalate and EDTA tubes respectively, where plasma blood samples were separated by centrifuge for glucose and insulin analyses and immediately frozen at −72 °C. The blood samples were stored at the University of Toronto, Toronto, Canada, until study completion. Mount Sinai Hospital, Pathology and Laboratory Medicine, Toronto, Canada, performed plasma glucose and insulin analyses. Plasma glucose was measured with Roche/Hitachi MODULAR P (Roche Diagnostics, Indianapolis, IN, USA) analyzer using the hexokinase method [29,30]. Plasma insulin was measured with MODULAR ANALYTICS E170 (Roche Diagnostics, Indianapolis, IN, USA) immunoassay analyzer and electrochemiluminescence immunoassay kit [31].

### 2.7. Calculations

Our pre-specified data plan called for two approaches to improve the precision of the analyses. First, the −30-min and 0-min glucose and insulin samples were pooled to provide a single fasting measurement (0-min). Second, although separate analyses were conducted for fructose and allulose, the data for the two controls (0 g fructose and 0 g allulose) were pooled for all comparisons.

Plasma glucose and insulin curves were plotted as incremental change over time. Total AUC and iAUC for plasma glucose and insulin were calculated for each participant geometrically, ignoring areas below the fasting value [32]. The Matsuda ISI_OGTT,_ a measure of insulin sensitivity, was calculated using the 75 g-OGTT plasma glucose (PG) and plasma insulin (PI) outcome: 10,000/√([FPG × FPI] × ([PG_MEAN_ × PI_MEAN_]), where PG is expressed in mg/dL (0.0555 mmol/L) and PI in μU/mL (6 pmol/L) [33]. The early insulin secretion index (∆PI_30-0_/∆PG_30-0_) was calculated as the change in PI from 0-min to 30-min divided by the change in PG over the same period [34]. The ISSI-2 is an OGTT-derived measure of β-cell function, calculated by the product of the ratio: total iAUC insulin/total iAUC glucose and the Matsuda ISI_OGTT._

### 2.8. Statistical Analyses

Statistical analyses were performed using STATA 13.1 (StataCorp LP, College Station, TX, USA). We recruited 25 participants to achieve a final sample size of *n* = 20 (based on 20% attrition) to detect a difference in iAUC plasma glucose of 60 mmol·min/L (based on a 20% reduction from 331 mmol·min/L assuming a standard deviation of 161 mmol·min/L with 90% power (1−β = 90%) [27]. The sample size also provided 80% power (1−β = 80%) to detect equivalence in the iAUC plasma glucose differences between fructose and allulose using margins (±δ) set at ±20% [35] and an estimated intra-subject standard deviation of 16.25% [27]. The 20% difference and equivalence margins were based on the minimal important difference proposed by Health Canada to support postprandial blood glucose response reduction claims [35]. The intra-individual coefficient of variation (CV) was calculated for the primary endpoint of plasma glucose iAUC using the following equation %CV = 100% × (SD/mean).

Separate analyses were conducted for fructose and allulose with the data averaged for the two controls (0 g) for comparisons with the two other doses (5 g, 10 g). Linear mixed-effects models were used to assess differences in all outcome measures with unstructured covariance for repeated measures within subjects. Although we had pre-specified using repeated measures ANOVA with the Dunnett’s test to adjust for the pairwise comparisons between each dose (5 g, 10 g) and the mean of the two controls (0 g) for fructose and allulose, we selected linear mixed-effects models as they allowed for the handling of missing data, fitting of the correlation between repeated measures in the same subject, and modeling of time effects [36,37]. We assessed the interactive effects of treatment and time (0, 30, 60, 90 and 120 min) on mean incremental changes in plasma glucose and insulin. To reduce the false discovery rate, the secondary and exploratory outcome measures were evaluated at *p* < 0.0125. We determined this alpha level by dividing α = 0.05/4, due to the four broad domains of our secondary and exploratory outcomes (glucose response, insulin sensitivity, insulin resistance, and insulin secretion) within which results would be expected to be correlated. All data are presented as mean ± standard error of the mean (SEM), unless specified otherwise.

Linear dose-response relationships were assessed using a continuous exposure variable in the mixed-effects model, while departures from linearity were assessed by comparing the linear dose model with the categorical dose model using a likelihood ratio test.

Subgroup analyses were conducted using linear mixed-effects models with interaction terms. We explored subgroup effect modification by age, sex, self-reported ethnicity, BMI, baseline HbA1c, fasting plasma glucose, fasting plasma insulin, and HOMA-IR.

Sensitivity analyses were performed to explore the influence of using the randomly assigned ‘allulose’ and ‘fructose’ controls (75 g-OGTT) in the analysis of all outcomes as compared to the methods of the main analysis, which used the pooled ‘fructose’ and ‘allulose’ controls. The sensitivity analyses were executed using the same linear mixed-effects models approach described above for the main analysis of all primary, secondary, and exploratory outcomes.

Equivalence testing was conducted, by assessing whether the upper and lower bounds of the 90% CI for the effect of allulose on iAUC for plasma glucose fell within the equivalence margins (± δ) set at ±20% using the two one sided tests (TOST) where the CI = (1−2α) × 100% at a significance level of α = 0.05. [35].

## 3. Results

### 3.1. Flow of Participants

Figure S1 presents the CONSORT diagram for the participants [38]. Recruitment took place from November 2015 to July 2016. Of the 53 individuals screened, 27 participants were randomized. There were two drop-outs after randomization, one subject refused to participate and one subject could not participate due to a conflict with work. Twenty-five subjects completed the trial.

### 3.2. Participant Characteristics

Table 1 represents the baseline characteristics of the 25 healthy participants of the Fructose and Allulose Catalytic Effects (FACE) trial. The 25 (13 male, 12 female) healthy volunteers in the FACE trial had an average age of 37 ± 16 years with a body mass index (BMI) of 24.7 ± 3.4 kg/m^2^. Participants were excluded if they; reported any prediabetes, diabetes, or other chronic diseases; and if they were smokers or heavy drinkers (>3 alcoholic beverages/day).

### 3.3. Primary Outcome: Glucose iAUC

**Figure 1:**
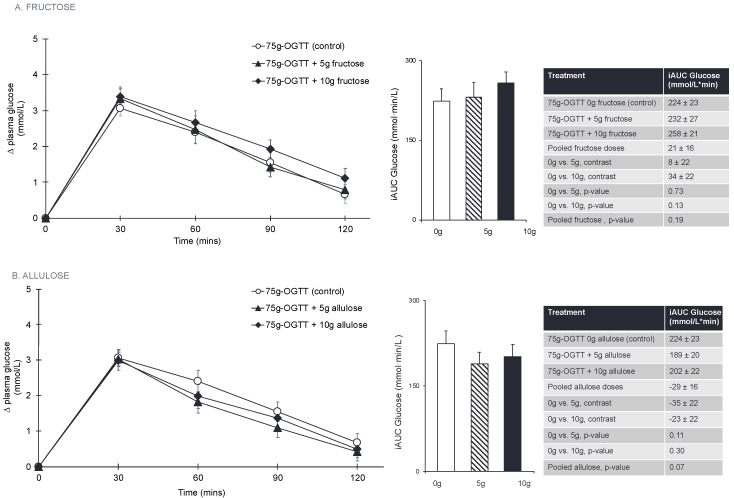
(**A**) Effect of small doses (5 g and 10 g) of fructose on incremental change in plasma glucose and the primary outcome incremental area under the curve (iAUC) for plasma glucose following consumption of 75 g-OGTT (control), 75 g-OGTT + 5 g fructose and 75 g-OGTT + 10 g fructose in 25 healthy participants. (**B**) Effect of small doses (5 g and 10 g) of allulose on incremental change in plasma glucose and the primary outcome of incremental area under the curve (iAUC) for plasma glucose following consumption of 75 g-OGTT (control), 75 g-OGTT + 5 g allulose and 75 g-OGTT + 10 g allulose in 25 healthy participants. Data reported as mean ± SEM. *p* < 0.05 was considered significant. Note that 5–10 g of fructose is 1–2% of total daily calories and allulose given at doses of 5–10 g contributes 0.05–0.1% of total daily calories in a 2000 kcal diet.

Figure 1A and Figure S2A present the effect of fructose on the postprandial blood glucose responses to a 75 g-OGTT. Pairwise comparisons did not show any significant effect of fructose on plasma glucose iAUC at any dose or when doses were pooled (*p* > 0.05). There was no linear (*p* = 0.13) or non-linear (*p* = 0.63) dose-response relationship between fructose dose and plasma glucose iAUC.

Figure 1B and Figure S2B present the effect of allulose at 0 g (control), 5 g, and 10 g on the postprandial blood glucose response to a 75 g-OGTT. Pairwise comparisons did not show any significant effects of allulose at any dose or when doses were pooled on plasma glucose iAUC (*p* > 0.05). There was no linear (*p* = 0.31) or non-linear (*p* = 0.22) dose-response relationship between allulose dose and plasma glucose iAUC.

### 3.4. Secondary Outcomes and Exploratory Outcome Measures

**Figure 2:**
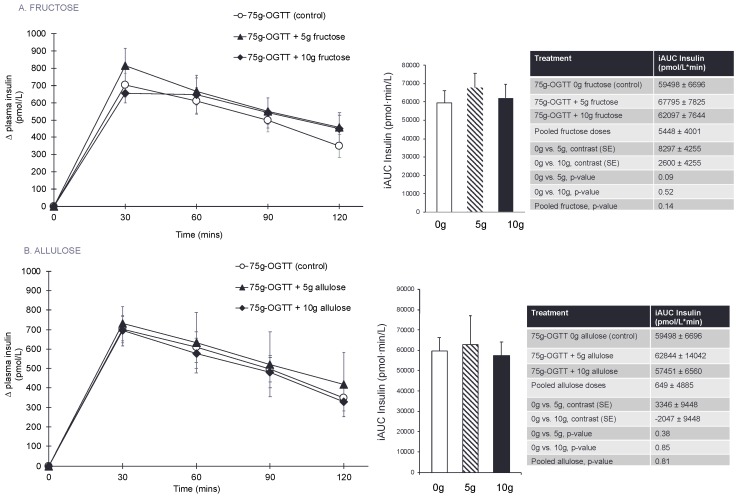
(**A**) Effect of small doses (5 g and 10 g) of fructose on the secondary outcomes of incremental change and incremental area under the curve (iAUC) for plasma insulin following consumption of 75 g-OGTT (control), 75 g-OGTT + 5 g fructose and 75 g-OGTT + 10 g fructose in 25 healthy participants. (**B**) Effect of small doses (5 g and 10 g) of allulose on the secondary outcomes of incremental change and incremental area under the curve (iAUC) for plasma insulin following consumption of 75 g-OGTT (control), 75 g-OGTT + 5 g allulose and 75 g-OGTT + 10 g allulose in 25 healthy participants. Data reported as mean ± SEM. Note that all *p*-values are from log-transformed data. *p* < 0.0125 was considered significant, see Section 2.8. Note that 5–10 g of fructose is 1–2% of total daily calories and allulose given at doses of 5–10 g contributes 0.05–0.1% of total daily calories in a 2000 kcal diet.

Figure 2A, Table 2, and Figures S3–S5 show the effect of fructose on the 75 g-OGTT derived secondary and exploratory outcome measures. Pairwise comparisons did not show a significant effect of fructose and no significant linear or non-linear dose responses were identified for any of the secondary or exploratory outcome measures (*p* > 0.0125).

Figure 2B, Table 3, and Figures S6–S8 show the effect of allulose on the 75 g-OGTT derived secondary and exploratory outcome measures. Pairwise comparisons did not show a significant effect of allulose and no significant linear or non-linear dose responses were identified for any of the secondary or exploratory outcome measures (*p* > 0.0125).

### 3.5. Subgroup Analyses

Figures S9 and S10 present the subgroup analyses for fructose and allulose, respectively. For the effect of fructose on plasma glucose iAUC, self-reported ethnicity was a significant effect modifier (*p* = 0.04). The significant effect was driven by three individuals who self-reported their ethnic category as ‘other’, which includes Middle Eastern, Latin American, and African American/Latin American. For allulose, there was no significant effect modification by any other subgroups (*p* > 0.05).

### 3.6. Sensitivity Analyses

Table S1 and Figures S11A, S12A, S13A, S14–S16 present the sensitivity analyses for the effect of fructose on the primary, secondary, and exploratory outcomes in response to a 75 g-OGTT in which the assigned 0 g-fructose control was used for all analyses. Contrary to our predictions, fructose lead to a significant increase in plasma glucose iAUC with the 10 g dose (*p* = 0.01) and with pooled fructose doses (*p* = 0.02). There was a significant increase in plasma insulin iAUC with the 5 g fructose dose (*p* = 0.01). There was no linear (*p* = 0.014) or non-linear (*p* = 0.88) dose-response relationship between fructose dose and plasma glucose iAUC. There was a significant, positive linear dose response relationship for the effect of fructose dose on mean incremental glucose (*p* = 0.008). There was a significant increase in both; incremental plasma glucose with the 10 g fructose dose (*p* = 0.01); and incremental plasma insulin with the 5 g fructose dose (*p* = 0.01).

Table S2 and Figures S11B, S12B, S13B, S17–S19 present the sensitivity analyses for the effect of allulose on the primary, secondary, and exploratory outcomes in response to a 75 g-OGTT in which the assigned 0 g-allulose control was used for all analyses. There was a significant decrease in plasma glucose iAUC with the 5 g allulose dose (*p* = 0.03). There was no linear (*p* = 0.10) or non-linear (*p* = 0.14) dose-response relationship between allulose dose and plasma glucose iAUC. Pairwise comparisons did not show significant effects of allulose at either dose or when doses were pooled (*p* > 0.0125) for any other secondary or exploratory endpoints. There were no significant linear or non-linear dose response relationships identified for any of the secondary or exploratory outcome measures (*p* > 0.0125).

### 3.7. Equivalence Testing

**Figure 3:**
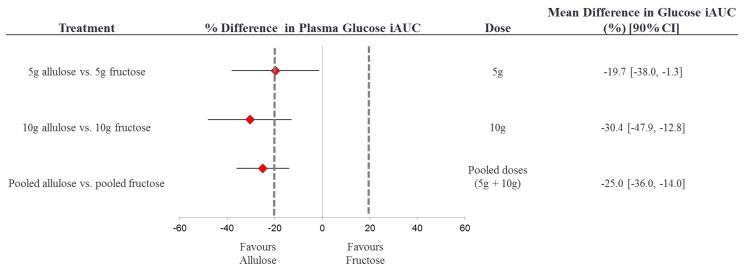
Equivalence test comparing the effect of allulose to fructose on plasma glucose iAUC. % difference plasma glucose iAUC = [(allulose_iAUCglucose_/control_iAUCglucose_) – (fructose_iAUCglucose_/control_iAUCglucose_)] × 100%. The dotted lines represent the ± 20% equivalence margins. The red diamond represents the mean difference and the black line crossing through the diamond represents the 90% CI. CI, confidence interval; iAUC, incremental area under the curve.

Figure 3 presents the results of the equivalence test between the effects of fructose and allulose on the primary endpoint of plasma glucose iAUC. The 90% CI for the effect of allulose compared to fructose (with fructose as the reference) on plasma glucose iAUC was inconclusive with the 5 g dose, 10 g dose, and when the doses were pooled since all 90% CI’s crossed the *a priori* equivalence margins of ±20% [35]. The 90% CI at all dose levels tended to favour allulose more than fructose, meaning that allulose showed a stronger trend for reduction in glucose iAUC as compared to fructose, although allulose was not superior to fructose.

Figure S20 presents the results of the equivalence test between the effects of fructose and allulose on the primary endpoint of plasma glucose iAUC in which the assigned 0 g fructose and 0 g allulose controls were used in a sensitivity analysis. The 90% CI for the effect of allulose compared to fructose (with fructose as the reference) on plasma glucose iAUC was inconclusive with the 5 g dose, 10 g dose, and when the doses were pooled since all 90% CI’s crossed the a priori equivalence margins of ±20% [35]. The 90% CI at all dose levels tended to favour allulose more than fructose, meaning that allulose showed a stronger trend for reduction in glucose iAUC as compared to fructose, although allulose was not superior to fructose.

### 3.8. Side Effects

Table S3 presents the side effects reported by participants. Participants tolerated the treatment drinks well overall. There were six reports of lightheadedness, five reports of mild nausea, and one report of fainting. For five of the participants who reported feeling unwell, the episodes of lightheadedness and mild nausea were brief, lasting for no longer than five to ten minutes after ingestion of the study drink. This is likely due to the high amount of sugar (75 g glucose, with or without 0 g, 5 g, or 10 g of additional allulose or fructose) consumed within a short (5-min) period of time. One participant, who accounted for five of six reports of lightheadedness, reported feeling unwell for a few hours following each treatment. In these cases, the issue was thought to be with the treatment protocol of the fasting blood sampling in combination with the study drink consumed in a short period of time, more than any one treatment in particular. 

## 4. Discussion

The FACE trial involving 25 healthy adults showed that small doses of fructose and allulose did not significantly affect postprandial blood glucose or markers of insulin secretion and sensitivity in response to a 75 g-OGTT.

Although the direction and magnitude of the effect seen in this trial are comparable to previous studies, we were unable to detect significance due to a high degree of within-subject variability in the glucose data. There was a non-significant 50 mmol·min/L difference between the glucose iAUC for the randomly assigned ‘allulose’ control (0 g-allulose + 75 g-OGTT) and the ‘fructose’ control (0 g-fructose + 75 g-OGTT), which translates to a 20% difference in participants’ responses to the same control drink (75 g-OGTT). The intra-individual (day-to-day) variation in plasma glucose iAUC that we observed was a coefficient of variation (CV) of 39%, which is considered high since normal subjects typically have a CV of 25% [39]. Normal subjects tend to have a more variable intra-individual glycemic response to carbohydrate (CV of ~25%) than those with type 2 diabetes (CV of ~15%), but there is also evidence that lean and obese normal subjects may have different glycemic responses [39]. For example, Wolever et al. [40,41] administered 4 repeated 75 g-OGTT’s to 19 normal subjects (*n* = 10, lean and *n* = 9, obese), where lean normal subjects had a CV of 39% and obese subjects had a CV of 26%. Our results align closely to the results of this study, as most of our subjects were fairly lean (mean BMI = 24.7 kg/m^2^) and our CV was 39%. Since the inclusion criteria for the FACE trial included healthy subjects with a BMI of 18.5–30 kg/m^2^, we anticipated a CV of 25% as this estimate includes both lean and obese normal individuals.

The high intra-individual variation in glucose responses to the control (75 g-OGTT) lead us to conduct sensitivity analyses, where the assigned ‘allulose’ and ‘fructose’ controls were used in the analysis of all outcomes, instead of the pooled controls we used in the main analysis. In the sensitivity analysis, we observed a significant reduction in plasma glucose iAUC due to the 5 g allulose treatment (*p* = 0.03). The results of the sensitivity analysis act to support our findings in the main analysis by illustrating the potential to find significant reductions in glucose iAUC with catalytic doses of allulose when there is less variation in the glucose responses to the control.

Additionally, since the study population was healthy, free of major disease, no diabetes, and relatively young (average age 37-y), their glucose homeostasis mechanisms are likely strong enough to resist major perturbations that would occur due to catalytic doses of fructose and allulose [42,43].

### 4.1. Results in the Context of Previous Studies

Although the reductions we observed were not significant, our results are largely in agreement with those previously reported for allulose. Iida et al. [20] found that doses ≥5 g of allulose significantly reduced postprandial plasma glucose iAUC by 22–32% and insulin iAUC by 28–31% in 20 healthy adults. Hayashi et al. [19] found that 5 g allulose significantly reduced postprandial glucose iAUC by 11% in 26 participants composed of both healthy participants (*n* = 11) and those classified as having borderline diabetes (*n* = 15). When authors stratified the results by healthy and borderline diabetes participants, they found a significant reduction of 13% in the participants with borderlines diabetes and a non-significant reduction in healthy individuals of 7% for plasma glucose iAUC. These patterns of reductions are similar—in magnitude and direction- to the reductions we observed in 25 healthy participants for our primary outcome of postprandial plasma glucose iAUC (16%).

On the other hand, we failed to confirm a ‘catalytic’ effect of fructose. We did not replicate previous findings [21,22,23] and even found signs of effects opposite to what we had hypothesized. Although we used a similar participant population to previous acute feeding trials, it may be that the fructose treatment did not induce the ‘catalytic’ mechanism in a timely manner. One possible explanation is variation in the timing of administration of fructose with the carbohydrate meal as tested by Heacock et al. [23]. The authors found that pre-feeding 10 g fructose 60- and 30-min before consumption of an instant mashed potato meal lead to a 25–27% reduction in glucose iAUC, but pairing 10 g fructose simultaneously with the instant mashed potato meal did not significantly reduce glucose iAUC. Perhaps ‘priming’ the catalytic mechanism with a small dose of fructose 1-h or 30-min before the consumption of the high carbohydrate load (i.e., 75 g-OGTT) is necessary for fructose-induced glycogen synthesis that leads to reductions in the postprandial blood glucose and insulin responses. In future investigations, it would be of interest to perform the same protocol as this trial with the modification of administering fructose at 1-h, 30-min, and 0-min before ingestion of the carbohydrate load.

While our study and the study by Heacock et al. [23] are both double-blinded, randomized crossover studies investigating the effect of catalytic doses of fructose on postprandial carbohydrate metabolism in healthy adults, there are several design differences between these studies that may have contributed to the difference in results. Firstly, Heacock et al. [23] had more study participants (*n* = 31) than our study (*n* = 25), which could have increased the power of their analysis. As well, we used a 75 g-OGTT as the carbohydrate load, whereas Heacock et al. [23] used an instant mashed potato meal (61.1 g) that contained 50 g of available carbohydrate, so the inherent differences in meal form and available carbohydrate content could have also contributed to the differential effects we observed. There was also a difference in blood sampling method where Heacock et al. [23] used finger-prick capillary whole blood glucose and then estimated plasma glucose using an AC analyzer, while our study took venous blood samples that were collected in fluoride oxalate tubes for plasma glucose collection. It is evident these methods of plasma glucose measurement are not directly comparable [44,45]. Lastly, our aim was to reproduce previous studies of fructose and allulose that investigated postprandial carbohydrate metabolism alone, whereas Heacock et al. [23] investigated *both* pre-feeding and simultaneous administration of catalytic fructose and the resultant effects on postprandial carbohydrate metabolism.

An interaction with ethnicity may have also contributed to the lack of a ‘catalytic’ effect of fructose. The subgroup analyses indicated that ethnicity was a significant effect modifier for the effect of fructose on plasma glucose iAUC (*p* = 0.04), such that fructose had a slight adverse effect (glucose iAUC elevation) for those in the ‘other’ ethnic category (including: Middle Eastern, South American, and South American/African-American). It should be noted that the ‘other’ ethnic category was comprised of *n* = 3 participants, so this is likely to be spurious effect due to the small sample size. In contrast, fructose has a neutral effect on plasma glucose iAUC for those in the other ethnic categories (Caucasian, South Asian, East Asian, African-American, and Caribbean). There is evidence that glycemic responses to carbohydrates differ by ethnicity, most notably there have been differences reported between Asians and Caucasians [46,47]. Glycemic responses to an oral glucose load were: 29% higher in Asians compared to Caucasians [46], and 39% higher in Chinese compared to European participants [47]. Although we did not observe significant differential glycemic responses for Asians and Caucasians in the present study, future investigations may want to assess these ethnic populations separately.

### 4.2. Strengths and Limitations

Our study was designed to increase precision in many ways, resulting in a high quality acute feeding trial. First, it was a randomized, double-blind crossover trial, a design that is considered one of the highest forms of clinical evidence [48]. Secondly, in the analyses we took the mean of the two control treatments to increase the precision of our control results such that we would have a strong comparator for the treatment groups. Thirdly, we took the mean of the two fasting blood samples (−30-min and 0-min) to create a precise basis for our glucose and insulin iAUC calculations.

A major limitation of our study was the high intra-individual CV in our participants’ glucose responses to the control 75 g-OGTT, such that we were unable to replicate previous studies for the effects of ‘catalytic’ doses of fructose and allulose on postprandial blood glucose regulation [19,20,21,22,23]. The mean within-subject CV for the 75 g-OGTT (control) was higher than anticipated at 39%. According to ISO guidelines, any intra-individual CV greater than 25% is considered high and should be interpreted with caution, as it points to high within-subject variability in postprandial blood glucose responses to at least two repeated tests (on separate occasions) with the same 75 g-OGTT [49,50]. A post-hoc power analysis for the primary outcome of glucose iAUC found that we had only 53% power to detect a difference between fructose and allulose treatments. Therefore, we were underpowered in this study to detect a statistically significant difference between fructose and allulose treatments on the primary endpoint of glucose iAUC. Therefore, future studies should calculate their power analysis based on the CV of 39%, due to the higher than predicated intra-individual variation in glycemic response we observed.

Another limitation of our study is that we did not include a pre-feeding treatment for fructose and allulose, similar to the design in Heacock et al. [23]. Perhaps we would have observed significant effects for fructose and allulose if we had administered catalytic doses (5 g, 10 g) of fructose and allulose 1-h or 30-min before consumption of the 75 g-OGTT. Future studies should explore variations in the time of administration of fructose and allulose, especially since pre-feeding has not been tested with allulose.

## 5. Conclusions

Catalytic doses (5 g and 10 g) of fructose and allulose did not significantly affect postprandial blood glucose regulation in response to a 75 g-OGTT in 25 healthy adults. The lack of effect appears to be mainly due to the higher than expected intra-individual variation in glucose responses. Future trials may consider recruiting larger sample sizes when studying this population. As well, it would be of interest to explore the effect of a pre-feeding treatment in future trials investigating the catalytic effects of fructose and allulose on postprandial carbohydrate metabolism.

## Figures and Tables

**Table 1:** Characteristics of study participants.

Characteristics	Total, *n* = 25	Males, *n* = 13	Females, *n* = 12
Age (years)	37 ± 16	40 ± 15	35 ± 17
Weight (kg)	69.3 ± 13.9	78.3 ± 11.7	59.5 ± 8.4
BMI (kg/m^2^)	24.7 ± 3.4	25.7 ± 3.9	23.6 ± 2.6
SBP (mmHg)	116 ± 8	120 ± 9	112 ± 6
DBP (mmHg)	70 ± 8	68 ± 9	71 ± 8
WC (cm)	81.2 ± 11.4	87.8 ± 11.0	74.3 ± 7.1

Mean ± SD; SBP, systolic blood pressure; DBP, diastolic blood pressure; WC, waist circumference.

**Table 2:** Secondary and exploratory outcomes for the effects of fructose on postprandial carbohydrate metabolism.

Outcome Measures *	75 g-OGTT 0 g Fructose Control	75 g-OGTT+ 5 g Fructose	75 g-OGTT+ 10 g Fructose	5 g vs. Control, Contrast	10 g vs. Control, Contrast	Pooled Doses vs. Control	5 g vs. Control, *p*-Value ^†^	10 g, vs. Control, *p*-Value ^†^	Pooled Doses vs. Control, *p*-Value ^†^
**Secondary**									
Abs C_max_ PG (mmol/L)	8.6 ± 0.3	8.8 ± 0.3	8.8 ± 0.2	0.2 ± 0.3	0.2 ± 0.3	0.2 ± 0.2	0.67	0.91	0.25
T_max_PG (min) **	41.4 ± 2.5	40.8 ± 4.5	39.6 ± 3.3	−0.6 ± 4.3	−1.8 ± 4.3	−1.2 ± 2.8	0.89	0.68	0.67
Inc PG (mmol/L)	1.5 ± 0.2	1.6 ± 0.2	1.8 ± 0.2	0.07 ± 0.2	0.29 ± 0.2	0.2 ± 0.1	0.68	0.07	0.14
iAUC PI (pmol·min/L)	59,498 ± 6696	67,795 ± 7825	62,097 ± 7644	8297 ± 4255	2600 ± 4255	5448 ± 4001	0.09	0.52	0.14
Abs C_max_ PI (pmol/L)	894 ± 79	976 ± 105	887 ± 107	82 ± 68	−7 ± 68	37 ± 56	0.45	0.46	0.82
T_max_ PI (min) **	49.8 ± 4.2	43.2 ± 3.9	54.0 ± 5.5	−6.6 ± 5.9	4.2 ± 5.9	−1.2 ± 4.1	0.26	0.47	0.77
Inc PI (pmol/L)	431 ± 51	498 ± 60	458 ± 58	66 ± 32	27 ± 32	47 ± 31	0.04	0.40	0.10
∆PI_30-0_/∆PG_30-0_	316 ± 66	357 ± 76	214 ± 23	42 ± 73	−101 ± 73	−30 ± 71	0.70	0.12	0.81
Matsuda ISI_OGTT_	3.9 ± 0.5	3.9 ± 0.6	3.6 ± 0.4	0.05 ± 0.3	−0.28 ± 0.3	−0.1 ± 0.2	0.45	0.34	0.45
**Exploratory**									
Total AUC PG (mmol·min/L)	854 ± 27	864 ± 31	880 ± 24	10 ± 22	26 ± 22	18 ± 16	0.65	0.24	0.26
Inc C_max_ PG (mmol/L)	3.3 ± 0.2	3.5 ± 0.3	3.7 ± 0.2	0.2 ± 0.3	0.3 ± 0.3	0.2 ± 0.2	0.54	0.22	0.16
Abs mean PG (mmol/L)	6.8 ± 0.2	6.9 ± 0.2	7.0 ± 0.2	0.1 ± 0.2	0.2 ± 0.2	0.1 ± 0.1	0.61	0.21	0.25
Total AUC Insulin (pmol·min/L)	68,192 ± 7201	75,494 ± 8358	70,241 ± 7909	7302 ± 4436	2049 ± 4436	4675 ± 4313	0.18	0.57	0.26
Inc C_max_ PI (pmol/L)	821 ± 75	911 ± 101	818 ± 105	90 ± 67	−3 ± 67	44 ± 53	0.34	0.48	0.70
Abs mean PI (pmol/L)	504 ± 55	562 ± 65	527 ± 60	58 ± 34	23 ± 34	40 ± 33	0.17	0.41	0.21
ISSI-2	289 ± 27	305 ± 25	275 ± 16	16 ± 18	−14 ± 18	0.8 ± 14	0.23	0.64	0.47

Data reported as mean ± SEM. Mean ± SEM and *p*-values are reported for *n* = 25. A linear mixed effect model was used to assess the differences in all outcome measures with unstructured covariance for repeated measures within subjects. * *p*-values were considered significant if *p* < 0.125, see Section 2.8. ** Data are skewed but all standard transformations were unable to remedy the issue, so data analyzed in raw form. † *p*-values for iAUC and total AUC PI, absolute and incremental C_max_ PI, absolute and incremental mean PI, ∆PI_30-0_/∆PG_30-0_, Matsuda ISI_OGTT_ and ISSI-2 correspond to log-transformed data due to non-normal distributions of residuals. Abs, absolute; C_max_, maximum concentration; iAUC, incremental area under the curve; Inc, incremental; ISSI-2, insulin secretion-sensitivity index-2; Matsuda ISI_OGTT_, Matsuda whole body insulin sensitivity index; PG, plasma glucose; PI, plasma insulin; tAUC, total area under the curve; T_max_, time of maximum concentration; ∆PI_30-0_/∆PG_30-0_, early insulin secretion index.

**Table 3:** Secondary and exploratory outcomes for the effects of allulose on postprandial carbohydrate metabolism.

Outcome Measures *	75 g-OGTT 0 g Allulose (Control)	75 g-OGTT+ 5 g Allulose	75 g-OGTT+ 10 g Allulose	5 g vs. Control, Contrast	10 g vs. Control, Contrast	Pooled Doses vs. Control, Contrast	5 g vs. Control, *p*-Value ^†^	10 g, vs. Control, *p*-Value ^†^	Pooled Doses vs. Control, *p*-Value ^†^
**Secondary**									
Abs C_max_ PG (mmol/L)	8.6 ± 0.3	8.4 ± 0.3	8.5 ± 0.3	−0.2 ± 0.3	−0.2 ± 0.3	−0.2 ± 0.2	0.44	0.53	0.30
T_max_PG (min) **	41.4 ± 2.5	33.6 ± 2.6	37.2 ± 3.1	−7.8 ± 3.5	−4.2 ± 3.5	−6.0 ± 2.7	0.03	0.23	0.03
Inc PG (mmol/L)	1.5 ± 0.2	1.3 ± 0.2	1.4 ± 0.2	−0.3 ± 0.2	−0.2 ± 0.2	−0.2 ± 0.1	0.11	0.30	0.05
iAUC PI (pmol·min/L)	59,498 ± 6696	62,844 ± 14042	57,451 ± 6560	3346 ± 9448	−2047 ± 9448	649 ± 4885	0.38	0.85	0.81
Abs C_max_ PI (pmol/L)	894 ± 79	957 ± 163	898 ± 82	63 ± 116	4 ± 116	34 ± 63	0.64	0.94	0.84
T_max_ PI (min) **	49.8 ± 4.2	40.8 ± 3.4	48.0 ± 3.9	−9.0 ± 3.6	−1.8 ± 3.6	−5.4 ± 3.3	0.01φ	0.62	0.13
Inc PI (pmol/L)	431 ± 51	461 ± 110	416 ± 50	29 ± 75	−16 ± 75	7 ± 37	0.36	0.83	0.80
∆PI_30-0_/∆PG_30-0_ θ	316 ± 66	374 ± 129	217 ± 87	58 ± 127	−99 ± 127	−21 ± 94	0.93	0.34	0.77
Matsuda ISI_OGTT_	3.9 ± 0.5	4.4 ± 0.7	3.7 ± 0.4	0.5 (0.3)	−0.2 (0.3)	0.1 ± 0.2	0.92	0.73	0.77
**Exploratory**									
tAUC PG (mmol·min/L)	854 ± 27	821 ± 24	827 ± 24	−33 ± 25	−26 ± 25	−30 ± 17	0.18	0.29	0.07
Inc C_max_ PG (mmol/L)	3.3 ± 0.2	3.1 ± 0.2	3.2 ± 0.2	−0.2 (0.2)	−0.1 (0.2)	−0.2 ± 0.17	0.35	0.56	0.26
Abs mean PG (mmol/L)	6.8 ± 0.2	6.6 ± 0.2	6.6 ± 0.2	−0.2 (0.2)	−0.2 (0.2)	−0.2 ± 0.1	0.19	0.28	0.07
tAUC Insulin (pmol·min/L)	68,192 ± 7201	72,734 ± 14510	66,174 ± 6805	4541 ± 9617	−2018 ± 9617	1262 ± 4786	0.45	0.99	0.96
Inc C_max_ PI (pmol/L)	821 ± 75	874 ± 158	825 ± 82	53 ± 115	4 ± 115	28 ± 63	0.63	0.97	0.90
Abs mean PI (pmol/L)	504 ± 55	543 ± 113	489 ± 52	39 ± 76	−15 ± 76	12 ± 37	0.45	0.99	0.99
ISSI-2	289 ± 27	300 ± 28	306 ± 26	11 ± 18	16 ± 18	14 ± 12	0.62	0.42	0.26

Data reported as mean ± SEM. Mean ± SEM and *p*-values are reported for *n* = 25. A linear mixed effect model was used to assess the differences in all outcome measures with unstructured covariance for repeated measures within subjects. * *p*-values were considered significant if *p* < 0.125, see Section 2.8. φ This value is rounded from *p* = 0.014, which is not considered significant. θ Descriptive statistics are reported for *n* = 25, but analysis was performed after one missing value was generated (*n* = 24) in the log transformation of the data. ** Data are skewed but all standard transformations were unable to remedy the issue, so data analyzed in raw form. † *p*-values for iAUC and total AUC PI, absolute and incremental C_max_ PI, absolute and incremental mean PI, ∆PI_30-0_/∆PG_30-0_, Matsuda ISI_OGTT_ and ISSI-2 correspond to log-transformed data due to non-normal distributions of residuals. Abs, absolute; C_max_, maximum concentration; iAUC, incremental area under the curve; Inc, incremental; ISSI-2, insulin secretion-sensitivity index-2; Matsuda ISI_OGTT_, Matsuda whole body insulin sensitivity index; PG, plasma glucose; PI, plasma insulin; tAUC, total area under the curve; T_max_, time of maximum concentration; ∆PI_30-0_/∆PG_30-0_, early insulin secretion index.

## Supplementary Materials

The following are available online at http://www.mdpi.com/2072-6643/10/6/750/s1. Table S1: Primary, secondary, and exploratory outcomes for the effects of fructose on postprandial carbohydrate metabolism, using the assigned 0 g-fructose control in all analyses. Table S2: Primary, secondary, and exploratory outcomes for the effects of allulose on postprandial carbohydrate metabolism, using the assigned 0 g-fructose control in all analyses. Table S3: Self-reported adverse events showed no pattern associated with treatment type. Figure S1: CONSORT statement for the healthy participants in the FACE Trial. Figure S2: (A) Linear (left) and non-linear dose-response (right) analysis of the effect of small doses of fructose on plasma glucose iAUC. (B) Linear (left) and non-linear (right) dose-response analysis of the effect of small doses of allulose on plasma glucose iAUC. In the linear dose-response graphs, the inner line represents the predicted linear trend, while the two outer lines represent the 95% CIs. In the non-linear dose-response graphs, the center line represents the mean, while the two outer lines represent the 95% CIs. *p*-values were considered significant if *p* < 0.0125. Figure S3: Linear (left) and non-linear (right) dose-response analysis of the effect of small doses of fructose on absolute C_max_ glucose (A), T_max_ glucose (B), mean incremental glucose (C), insulin iAUC (D), absolute C_max_ insulin (E) and T_max_ insulin (F). In the linear dose-response graphs, the inner line represents the predicted linear trend, while the two outer lines represent the 95% CIs. In the non-linear dose-response graphs, the center line represents the mean, while the two outer lines represent the 95% CIs. *p*-values correspond to log-transformed data due to non-normal distribution of residuals for (D) and (E). *p*-values were considered significant if *p* < 0.0125. Figure S4: Linear (left) and non-linear (right) dose-response analysis of the effect of small doses of fructose mean incremental insulin (A), Early Insulin Secretion Index (∆PI_30-0_/∆PG_30-0_) (B), Matsuda Insulin Sensitivity Index_OGTT_ (C), total AUC glucose (D), incremental C_max_ glucose (E) and absolute mean glucose (F). In the linear dose-response graphs, the inner line represents the predicted linear trend, while the two outer lines represent the 95% CIs. In the non-linear dose-response graphs, the center line represents the mean, while the two outer lines represent the 95% CIs. *p*-values correspond to log-transformed data due to non-normal distribution of residuals for (A), (B), and (C). *p*-values were considered significant if *p* < 0.0125. Figure S5: Linear (left) and non-linear (right) dose-response analysis of the effect of small doses of fructose on total AUC insulin (A), incremental C_max_ insulin (B), absolute mean insulin (C) and Insulin Secretion-Sensitivity Index-2 (ISSI-2) (D). In the linear dose-response graphs, the inner line represents the predicted linear trend, while the two outer lines represent the 95% CIs. In the non-linear dose-response graphs, the center line represents the mean, while the two outer lines represent the 95% CIs. *p*-values correspond to log-transformed data due to non-normal distribution of residuals for (A), (B), (C), and (D). *p*-values were considered significant if *p* < 0.0125. Figure S6: Linear (left) and non-linear (right) dose-response analysis of the effect of small doses of allulose on absolute C_max_ glucose (A), T_max_ glucose (B), mean incremental glucose (C), insulin iAUC (D), absolute C_max_ insulin (E) and T_max_ insulin (F). In the linear dose-response graphs, the middle black line represents the predicted linear trend, while the two outer lines represent the 95% CIs. In the non-linear dose-response graphs, the middle black line represents the mean, while the two grey lines represent the 95% CIs. *p*-values correspond to log-transformed data due to non-normal distribution of residuals for (D) and (E). *p*-values were considered significant if *p* < 0.0125. Figure S7: Linear (left) and non-linear (right) dose-response analysis of the effect of small doses of allulose on mean incremental insulin (A), Early Insulin Secretion Index (∆PI_30-0_/∆PG_30-0_) (B), Matsuda Insulin Sensitivity Index_OGTT_ (C), total AUC glucose (D), incremental C_max_ glucose (E) and absolute mean glucose (F). In the linear dose-response graphs, the middle black line represents the predicted linear trend, while the two grey lines represent the 95% CIs. In the non-linear dose-response graphs, the middle black line represents the mean, while the two outer lines represent the 95% CIs. *p*-values correspond to log-transformed data due to non-normal distribution of residuals for (A), (B), and (C). *p*-values were considered significant if *p* < 0.0125. Figure S8: Linear (left) and non-linear (right) dose-response analysis of the effect of small doses of allulose on total AUC insulin (A), incremental C_max_ insulin (B), absolute mean insulin (C) and Insulin Secretion-Sensitivity Index-2 (ISSI-2) (D). In the linear dose-response graphs, the middle black line represents the predicted linear trend, while the two grey lines represent the 95% CIs. In the non-linear dose-response graphs, the middle black line represents the mean, while the two outer lines represent the 95% CIs. *p*-values correspond to log-transformed data due to non-normal distribution of residuals for (A), (B), (C) and (D). *p*-values were considered significant if *p* < 0.0125. Figure S9: Post-hoc subgroup analyses for the effect of fructose on the difference in glucose iAUC between pooled doses and control. Data for HbA1c, fasting plasma glucose and insulin, and HOMA-IR are based on visit 1 data (baseline) for all subjects. The category ‘Other’ under ethnicity includes; Middle Eastern, South-American, and South-American/African-American. * For those from Asian descent, ethnic specific BMI cut-offs were used. Where BMI < 22.9 kg/m^2^ = normal, BMI 23–24.9 kg/m^2^ = overweight, and BMI > 25 kg/m^2^ = obese. For all other ethnicities, BMI < 24.9 kg/m^2^= normal, BMI ≥ 25–29.9 kg/m^2^ = overweight, and BMI > 30 kg/m^2^ = obese BMI, Body mass index; FPI, fasting plasma insulin; FPG, fasting plasma glucose; HbA1c, Glycated hemoglobin; HOMA-IR, homeostasis model assessment-insulin resistance. *p*-values < 0.0125 were considered significant. Figure S10: Post-hoc subgroup analyses for the effect of allulose on the difference in glucose iAUC between pooled doses and control. Data for HbA1c, fasting plasma glucose and insulin, and HOMA-IR are based on visit 1 data (baseline) for all subjects. The category ‘Other’ under ethnicity includes; Middle Eastern, South-American, and South-American/African-American. *For those of Asian descent, ethnic specific BMI cut-offs were used. Where BMI < 22.9 kg/m^2^ = normal, BMI 23–24.9 kg/m^2^ = overweight, and BMI > 25 kg/m^2^ = obese. For all other ethnicities, BMI< 24.9 kg/m^2^ = normal, BMI ≥ 25–29.9 kg/m^2^ = overweight, and BMI > 30 kg/m^2^ = obese. BMI, Body mass index; FPI, fasting plasma insulin; FPG, fasting plasma glucose; HbA1c, Glycated hemoglobin; HOMA-IR, homeostasis model assessment-insulin resistance. *p*-values < 0.0125 were considered significant. Figure S11: (A) Effect of small doses of fructose on incremental change and incremental area under the curve (iAUC) for plasma glucose following consumption of 75 g-OGTT (assigned 0 g fructose control), 75 g-OGTT + 5 g fructose and 75 g-OGTT + 10 g fructose in 25 healthy participants. (B) Effect of small doses of allulose on incremental change and incremental area under the curve (iAUC) for plasma glucose following consumption of 75 g-OGTT (assigned 0 g allulose control), 75 g-OGTT + 5 g allulose and 75 g-OGTT + 10 g allulose in 25 healthy participants. Data reported as mean + SEM. SE, standard error. **p*-values < 0.05 were considered significant. Figure S12: (A) Effect of small doses of fructose on incremental change and incremental area under the curve (iAUC) for plasma insulin following consumption of 75 g-OGTT (assigned 0 g fructose control), 75 g-OGTT + 5 g fructose and 75 g-OGTT + 10 g fructose in 25 healthy participants. (B) Effect of small doses of allulose on incremental change and incremental area under the curve (iAUC) for plasma insulin following consumption of 75 g-OGTT (assigned 0 g allulose control), 75 g-OGTT + 5 g allulose and 75 g-OGTT + 10 g allulose in 25 healthy participants. Data reported as mean ± SEM. SE, standard error. Note that all *p*-values are from log-transformed data due to non-normality of residuals. *p*-values < 0.0125 were considered significant. * *p*-value was rounded down from *p* = 0.014 and is not considered significant. Figure S13: (A) Linear (left) and non-linear dose-response (right) analysis of the effect of small doses of fructose on plasma glucose iAUC in a sensitivity analysis using the assigned fructose control. (B) Linear (left) and non-linear (right) dose-response analysis of the effect of small doses of allulose on plasma glucose iAUC in a sensitivity analysis using the assigned allulose control. In the linear dose-response graphs, the middle black line represents the predicted linear trend, while the two grey lines represent the 95% CIs. In the non-linear dose-response graphs, the middle black line represents the mean, while the two grey lines represent the 95% CIs. *p*-values were considered significant if *p* < 0.0125. Figure S14: Linear (left) and non-linear (right) dose-response analysis of the effect of small doses of fructose on absolute C_max_ glucose (A), T_max_ glucose (B), mean incremental glucose (C), insulin iAUC (D), absolute C_max_ insulin (E) and T_max_ insulin (F). This sensitivity analysis used the assigned fructose 0 g-control in the dose response analysis. In the linear dose-response graphs, the middle black line represents the predicted linear trend, while the two outer lines represent the 95% CIs. In the non-linear dose-response graphs, the middle black line represents the mean, while the two outer lines represent the 95% CIs. *p*-values correspond to log-transformed data due to non-normal distribution of residuals for (D) and (E). * *p*-values were considered significant if *p* < 0.0125. Figure S15: Linear (left) and non-linear (right) dose-response analysis of the effect of small doses of fructose mean incremental insulin (A), Early Insulin Secretion Index (∆PI_30-0_/∆PG_30-0_) (B), Matsuda Insulin Sensitivity Index_OGTT_ (C), total AUC glucose (D), incremental C_max_ glucose (E) and absolute mean glucose (F). This sensitivity analysis used the assigned fructose 0 g-control in the dose response analysis. In the linear dose-response graphs, the inner line represents the predicted linear trend, while the two outer lines represent the 95% CIs. In the non-linear dose-response graphs, the center line represents the mean, while the two outer lines represent the 95% CIs. *p*-values correspond to log-transformed data due to non-normal distribution of residuals for (A), (B), and (C). *p*-values were considered significant if *p* < 0.0125. Figure S16: Linear (left) and non-linear (right) dose-response analysis of the effect of small doses of fructose on total AUC insulin (A), incremental C_max_ insulin (B), absolute mean insulin (C) and Insulin Secretion-Sensitivity Index-2 (ISSI-2) (D). This sensitivity analysis used the assigned fructose 0 g-control in the dose response analysis. In the linear dose-response graphs, the inner line represents the predicted linear trend, while the two outer lines represent the 95% CIs. In the non-linear dose-response graphs, the center line represents the mean, while the two outer lines represent the 95% CIs. *p*-values correspond to log-transformed data due to non-normal distribution of residuals for (A), (B), (C), and (D). *p*-values were considered significant if *p* < 0.0125. Figure S17: Linear (left) and non-linear (right) dose-response analysis of the effect of small doses of allulose on absolute Cmax glucose (A), Tmax glucose (B), mean incremental glucose (C), insulin iAUC (D), absolute Cmax insulin (E) and Tmax insulin (F). This sensitivity analysis used the assigned allulose 0 g-control in the dose response analysis. In the linear dose-response graphs, the middle black line represents the predicted linear trend, while the two outer lines represent the 95% CIs. In the non-linear dose-response graphs, the middle black line represents the mean, while the two grey lines represent the 95% CIs. *p*-values correspond to log-transformed data due to non-normal distribution of residuals for (D) and (E). *p*-values were considered significant if *p* < 0.0125. Figure S18: Linear (left) and non-linear (right) dose-response analysis of the effect of small doses of allulose on mean incremental insulin (A), Early Insulin Secretion Index (∆PI_30-0_/∆PG_30-0_) (B), Matsuda Insulin Sensitivity Index_OGTT_ (C), total AUC glucose (D), incremental C_max_ glucose (E) and absolute mean glucose (F). This sensitivity analysis used the assigned allulose 0 g-control in the dose response analysis. In the linear dose-response graphs, the middle black line represents the predicted linear trend, while the two grey lines represent the 95% CIs. In the non-linear dose-response graphs, the middle black line represents the mean, while the two outer lines represent the 95% CIs. *p*-values correspond to log-transformed data due to non-normal distribution of residuals for (A), (B), and (C). *p*-values were considered significant if *p* < 0.0125. Figure S19: Linear (left) and non-linear (right) dose-response analysis of the effect of small doses of allulose on total AUC insulin (A), incremental C_max_ insulin (B), absolute mean insulin (C) and Insulin Secretion-Sensitivity Index-2 (ISSI-2) (D). This sensitivity analysis used the assigned allulose 0 g-control in the dose response analysis. In the linear dose-response graphs, the middle black line represents the predicted linear trend, while the two grey lines represent the 95% CIs. In the non-linear dose-response graphs, the middle black line represents the mean, while the two outer lines represent the 95% CIs. *p*-values correspond to log-transformed data due to non-normal distribution of residuals for (A), (B), (C) and (D). *p*-values were considered significant if *p* < 0.0125. Figure S20: Equivalence test comparing the effect of allulose to fructose on plasma glucose iAUC. % difference plasma glucose iAUC = [(allulose_iAUCglucose_/control_iAUCglucose_) – (fructose_iAUCglucose_/control_iAUCglucose_)] × 100%. The assigned 0 g fructose and 0 g allulose controls were used for this analysis. The dotted lines represent the ± 20% equivalence margins. The red diamond represents the mean difference and the black line crossing through the diamond represents the 90% CI. CI, confidence interval; iAUC, incremental area under the curve.
