# Supplementary material for: A Double-Blind, Randomized Controlled, Acute Feeding Equivalence Trial of Small, Catalytic Doses of Fructose and Allulose on Postprandial Blood Glucose Metabolism in Healthy Participants: The Fructose and Allulose Catalytic Effects (FACE) Trial
Source: Nutrients. 2018 Jun 9;10(6):750. doi: 10.3390/nu10060750 (PMC6024645; doi:10.3390/nu10060750)
Supplement: Supplementary file 1 [file nutrients-10-00750-s001.pdf]

Supplementary Materials: A Double-Blind, Randomized Controlled, Acute Feeding Equivalence Trial of Small, Catalytic Doses of Fructose and Allulose on Postprandial Blood Glucose Metabolism in Healthy Participants: The Fructose and Allulose Catalytic Effects (FACE) trial

Catherine R. Braunstein, Jarvis C. Noronha, Andrea J. Glenn, Effie Viguiliouk, Rebecca Noseworthy, Tauseef A. Khan, Fei Au-Yeung, Sonia Blanco Mejia, Thomas M.S. Wolever, Robert G. Josse, Cyril W.C. Kendall, John L. Sievenpiper

1. Supplementary Tables

Table S1. Secondary and exploratory outcomes for the effects of fructose on postprandial carbohydrate metabolism, using the assigned 0g-fructose control in all analyses.

Outcome Measures	75g-OGTT 0g fructose (control)	75g-OGTT+ 5g fructose	75g-OGTT+ 10g fructose	5g vs. control , contrast	10g vs. control, contrast	Pooled doses vs. control, contrast (SE)	5g vs. control, p-value†	10g vs. control, p-value†	Pooled doses vs. control, p-value†
**Secondary**									
**Abs C_{max} PG (mmol/L)**	8.4 ± 0.3	8.8 ± 0.3	8.8 ± 0.2	0.4 ± 0.3	0.5 ± 0.3	0.4 ± 0.3	0.20	0.14	0.10
**T_{max}PG (min)****	40.8 ± 3.4	40.8 ± 4.5	39.6 ± 3.3	-3.6e-15 ± 4.3	-1.2 ± 4.3	-0.6 ± 2.6	0.99	0.78	0.82
**Inc PG (mmol/L)**	1.4 ± 0.2	1.6 ± 0.2	1.8 ± 0.2	0.2 ± 0.2	0.5 ± 0.2	0.3 ± 0.1	0.18	0.01*	0.01ϕ
**iAUC PI (pmol·min/L)**	56299 ± 7336	67795 ± 7825	62097 ± 7644	11496 ± 4877	5798 ± 4877	8647 ± 4952	0.01ϕ	0.08	0.05
**Abs C_{max} PI (pmol/L)**	829 ± 86	976 ± 105	886 ± 107	146 ± 80	57 ± 80	102 ± 76	0.06	0.42	0.17
**T_{max} PI (min)****	50.4 ± 4.8	43.2 ± 3.9	54.0 ± 5.5	-7.2 ± 6.2	3.6 ± 6.2	-1.8 ± 4.9	0.25	0.56	0.71
**Inc PI (pmol/L)**	409 ± 56	498 ± 60	458 ± 58	89 ± 36	49 ± 36	69 ± 37	0.01*	0.05	0.03

**$\Delta PI_{30-0}/\Delta PG_{30-0}$ θ**	420 ± 136	357 ± 76	214 ± 23	-63 ± 94	-206 ± 94	-134 ± 102	0.91	0.04	0.41
**Matsuda ISI_{OGTT}**	4.0 ± 0.5	3.9 ± 0.6	3.6 ± 0.4	-0.02 ± 0.4	-0.3 ± 0.4	-0.2 ± 0.4	0.72	0.61	0.77
**Exploratory**									
**tAUC PG (mmol·min/L)**	834 ± 28	864 ± 30	880 ± 24	30 ± 24	46 ± 24	38 ± 21	0.21	0.06	0.06
**Inc C_{max} PG (mmol/L)**	3.1 ± 0.3	3.5 ± 0.3	3.7 ± 0.2	0.4 ± 0.3	0.6 ± 0.3	0.5 ± 0.2	0.17	0.05	0.05
**Abs mean PG (mmol/L)**	6.7 ± 0.2	6.9 ± 0.2	7.0 ± 0.2	0.2 ± 0.2	0.3 ± 0.2	0.3 ± 0.1	0.23	0.06	0.07
**tAUC Insulin (pmol·min/L)**	65639 ± 7997	75494 ± 8358	70241 ± 7909	9855 ± 5405	4603 ± 5405	7229 ± 5736	0.05	0.14	0.10
**Inc C_{max} PI (pmol/L)**	751 ± 79	911 ±101	818 ± 105	160 ± 76	67 ± 76	113 ± 70	0.04	0.31	0.12
**Abs mean PI (pmol/L)**	487 ± 61	562 ± 65	527 ± 60	75 ± 41	40 ± 41	57 ± 43	0.05	0.11	0.09
**ISSI-2**	282 ± 24	305 ± 25	275 ± 16	23 ± 17	-7 ± 17	8 ± 12	0.09	0.83	0.22

Data reported as mean ± SEM. Mean ± SEM and p-values are reported for n=25. A linear mixed effect model was used to assess the differences in all outcome measures with unstructured covariance for repeated measures within subjects. *P-values were considered significant if P<0.0125, see Section 2.8. ϕ Values were rounded down and are not significant. θ Descriptive statistics are reported for n=25, but analysis was performed after one missing value was generated (n=24) in the log transformation of the data. ** Data are skewed but all standard transformations were unable to remedy the issue, so data analyzed in raw form. † P-values for iAUC and total AUC PI, absolute and incremental C_{max} PI, absolute and incremental mean PI, $\Delta PI_{30-0}/\Delta PG_{30-0}$, Matsuda ISI_{OGTT} and ISSI-2 correspond to log-transformed data due to non-normal distributions of residuals. Abs, absolute; C_{max}, maximum concentration; iAUC, incremental area under the curve; Inc, incremental; ISSI-2, insulin secretion-sensitivity index-2; Matsuda ISI_{OGTT}, Matsuda whole body insulin sensitivity index; PG, plasma glucose; PI, plasma insulin; tAUC, total area under the curve; T_{max}, time of maximum concentration; $\Delta PI_{30-0}/\Delta PG_{30-0}$, early insulin secretion index.

Table S2. Secondary and exploratory outcomes for the effects of fructose on postprandial carbohydrate metabolism, using the assigned 0g-allulose control in all analyses.

Outcome Measures	75g-OGTT 0g allulose (control)	75g-OGTT+ 5g allulose	75g-OGTT+ 10g allulose	5g vs. control , contrast (SE)	10g vs. control, contrast (SE)	Pooled doses vs. control, contrast (SE)	5g vs. control, p-value†	10g, vs. control, p-value†	Pooled doses vs. control, p-value†
**Secondary**									
**Abs C_{max} PG (mmol/L)**	8.9 ± 0.3	8.4 ± 0.3	8.5 ± 0.3	-0.4 ± 0.3	-0.4 ± 0.3	-0.4 ± 0.3	0.19	0.23	0.15
**T_{max} PG (min) ****	42.0 ± 3.9	33.6 ± 2.6	37.2 ± 3.1	-8.4 ± 4.5	-4.8 ± 4.5	-6.6 ± 4.3	0.06	0.29	0.13
**Inc PG (mmol/L)**	1.7 ± 0.2	1.3 ± 0.2	1.4 ± 0.2	-0.4 ± 0.2	-0.3 ± 0.2	-0.4 ± 0.2	0.04	0.10	0.04
**iAUC PI (mmol·min/L)**	62696 ± 6853	62844 ± 14042	57451 ± 6560	147 ± 9652	-5246 ± 9652	-2549 ± 5451	0.19	0.50	0.72
**Abs C_{max} PI (pmol/L)**	958 ± 90	957 ± 163	898 ± 82	-1 ± 121	-60 ± 121	-31 ± 75	0.39	0.71	0.17
**T_{max} PI (min) ****	49.2 ± 5.4	40.8 ± 3.4	48.0 ± 3.9	-8.4 ± 4.5	-1.2 ± 4.5	-4.8 ± 4.6	0.06	0.79	0.30
**Inc PI (pmol/L)**	454 ± 51	461 ± 110	416 ± 50	7 ± 76	-38 ± 76	-16 ± 42	0.18	0.50	0.43
**$\Delta PI_{30-0}/\Delta PG_{30-0}$ θ**	278 ± 38	374 ± 129	217 ± 87	96 ± 113	-61 ± 113	17 ± 62	0.73	0.43	0.26
**Matsuda ISI_{OGTT}**	3.8 ± 0.5	4.4 ± 0.7	3.7 ± 0.4	0.6 ± 0.4	-0.2 ± 0.4	0.2 ± 0.3	0.62	0.82	0.39
**Exploratory**									
**tAUC PG (mmol·min/L)**	874 ± 36	821 ± 24	827 ± 24	-54 ± 31	-47 ± 31	-50 ± 29	0.09	0.14	0.08

**Inc C_{max} PG (mmol/L)**	3.6 ± 0.3	3.1 ± 0.2	3.2 ± 0.2	-0.5 ± 0.3	-0.4 ± 0.3	-0.4 ± 0.3	0.11	0.19	0.09
**Abs mean PG (mmol/L)**	6.9 ± 0.3	6.6 ± 0.2	6.6 ± 0.2	-0.4 ± 0.2	-0.3 ± 0.2	-0.4 ± 0.2	0.10	0.15	0.09
**tAUC Insulin (pmol/L*min)**	70746 ± 7348	72734 ± 14510	66174 ± 6805	1988 ± 9844	-4572 ± 9844	-1292 ± 5435	0.29	0.73	0.64
**Inc C_{max} PI (pmol/L)**	891 ± 87	874 ± 158	825 ± 82	-17 ± 119	-66 ± 119	-42 ± 73	0.33	0.57	0.60
**Abs mean PI (pmol/L)**	521 ± 55	543 ± 113	489 ± 52	22 ± 78	-32 ± 78	-5 ± 42	0.32	0.77	0.70
**ISSI-2**	297 ± 33	300 ± 28	306 ± 26	3 ± 22	9 ± 22	6 ± 20	0.79	0.60	0.53

Data reported as mean ± SEM. Mean ± SEM and p-values are reported for n=25. A linear mixed effect model was used to assess the differences in all outcome measures with unstructured covariance for repeated measures within subjects. P-values were considered significant if P<0.0125, see Section 2.8. θ Descriptive statistics are reported for n=25, but analysis was performed after one missing value was generated (n=24) in the log transformation of the data. ** Data are skewed but all standard transformations were unable to remedy the issue, so data analyzed in raw form. † P-values for iAUC and total AUC PI, absolute and incremental C_{max} PI, absolute and incremental mean PI, $\Delta PI_{30-0}/\Delta PG_{30-0}$, Matsuda ISI_{OGTT} and ISSI-2 correspond to log-transformed data due to non-normal distributions of residuals. Abs, absolute; C_{max}, maximum concentration; iAUC, incremental area under the curve; Inc, incremental; ISSI-2, insulin secretion-sensitivity index-2; Matsuda ISI_{OGTT}, Matsuda whole body insulin sensitivity index; PG, plasma glucose; PI, plasma insulin; tAUC, total area under the curve; T_{max}, time of maximum concentration; $\Delta PI_{30-0}/\Delta PG_{30-0}$, early insulin secretion index.

Table S3. Self-reported adverse events showed no pattern associated with treatment type.

Participant ID	Self-Reported Adverse Event	Treatment
**1 (dropout)**	The participant e-mailed study staff and indicated that he had decided not to complete the final study visit due to symptoms (mainly fatigue, dizziness, low BP) he experienced after the last (5th) study visit. Participant now considered study drop out.	75g-OGTT,<br>5g A+ 75g-OGTT,<br>10g A + 75g-OGTT, and<br>5g F + 75g-OGTT.
**2**	Participant felt light-headed after -30-min sample and laid down for 20-30 minutes. He felt lightheaded after 0-min sample but felt better after consuming OGTT.	5g F+ 75g-OGTT
**3**	Participant reported feeling nauseous post OGTT but this improved over time.	10g F + 75g-OGTT
**3**	Participant reported feeling nauseous after consuming the OGTT.	75g-OGTT
**4**	Participant felt nauseous after consuming the OGTT.	75g-OGTT
**4**	Participant felt nauseous after consuming the OGTT.	75g-OGTT
**5**	Direct venous blood draw was used for the final blood sample (as the catheter failed). Afterwards, participant felt unwell and asked to lie down. Participant laid down for 5 mins and then felt okay.	5g F + 75g OGTT
**6**	Participant informed study staff on visit 3 that participant fainted after visit 2. Participant did not inform study staff that he had consumed 4L of Peglyte for the purposes of a colonoscopy, the night before the clinic visit. Participant was scheduled to have a colonoscopy on the afternoon after visit 2 but missed it due to the fainting episode. Incident was reported to St. Michael's Hospital REB.	75g-OGTT

A, allulose; BP, blood pressure; F, fructose; REB, research ethics board; SAE, severe adverse event; OGTT, oral glucose

2. Supplementary Figures

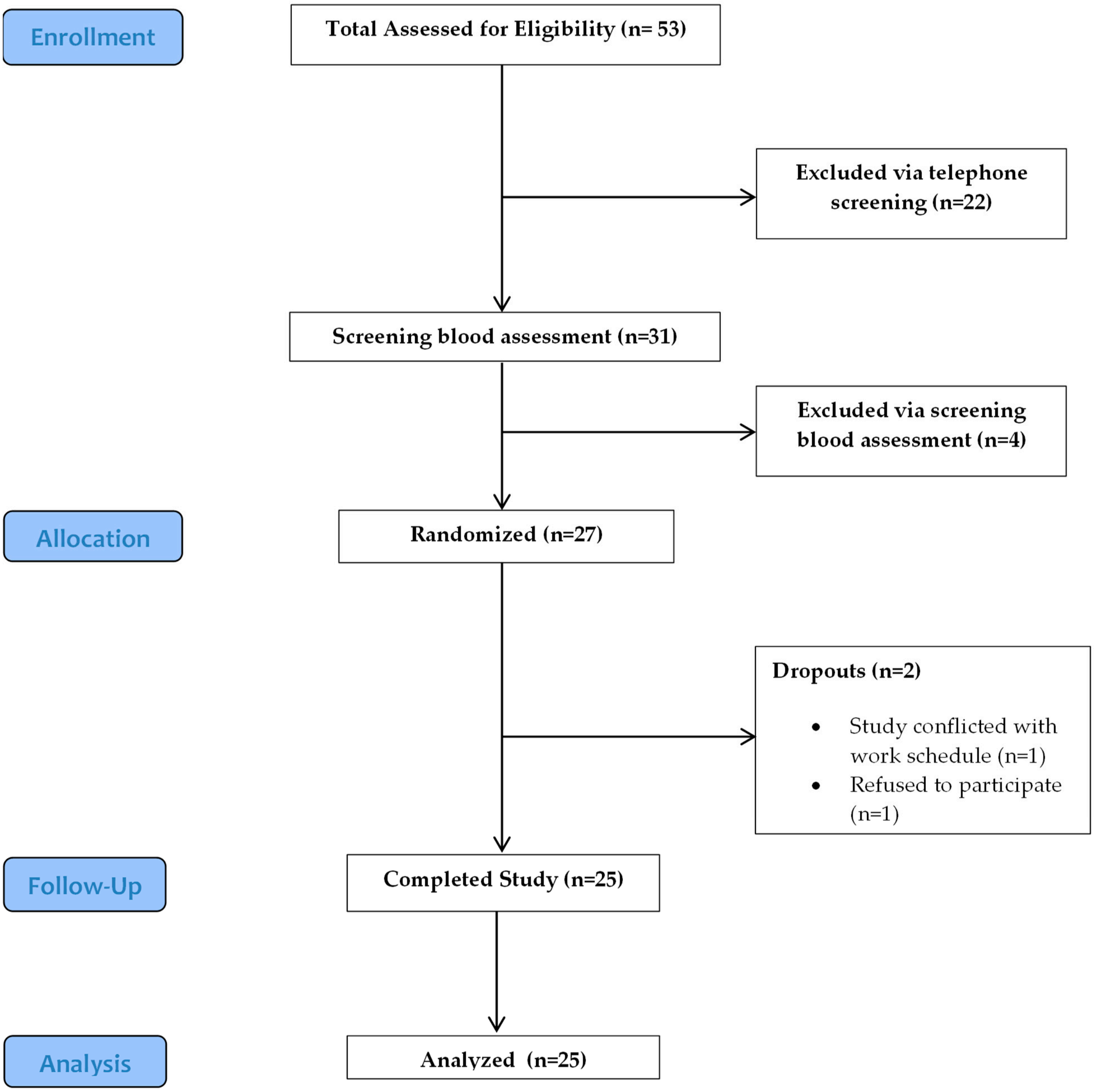

Figure S1. CONSORT statement for the healthy participants in the FACE Trial.

A. Fructose

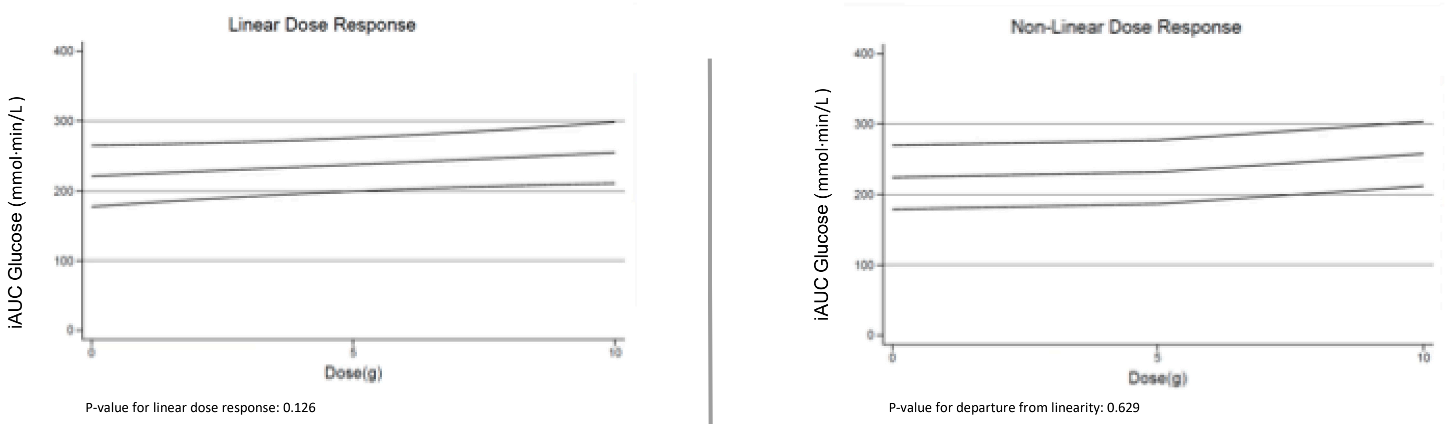

B. Allulose

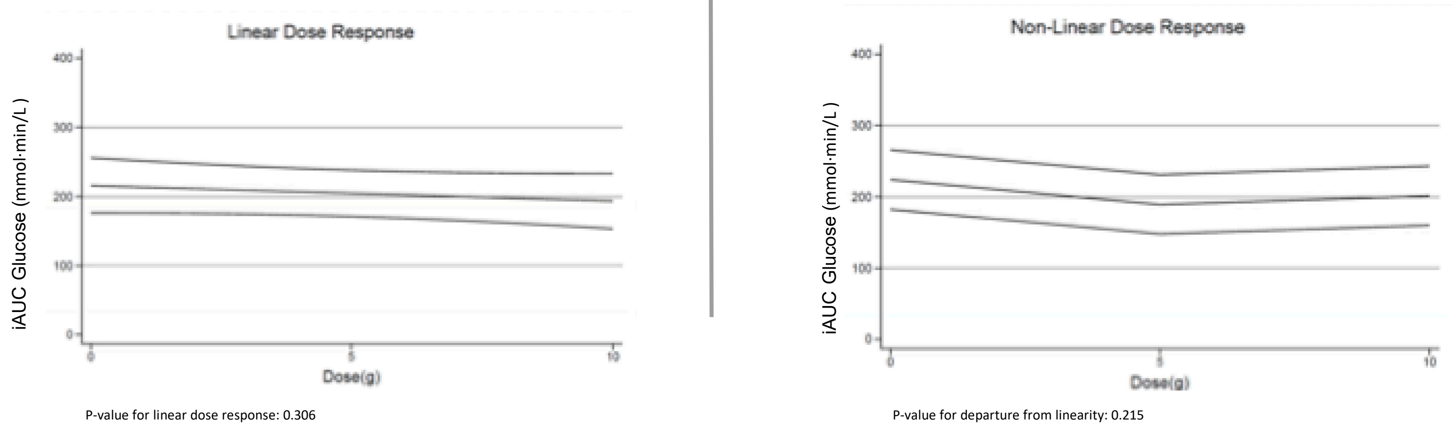

Figure S2. (A) Linear (left) and non-linear dose-response (right) analysis of the effect of small doses of fructose on plasma glucose iAUC. (B) Linear (left) and non-linear (right) dose-response analysis of the effect of small doses of allulose on plasma glucose iAUC. In the linear dose-response graphs, the inner line represents the predicted linear trend, while the two outer lines represent the 95% CIs. In the non-linear dose-response graphs, the center line represents the mean, while the two outer lines represent the 95% CIs. P-values were considered significant if $P<0.0125$, see Section 2.8.

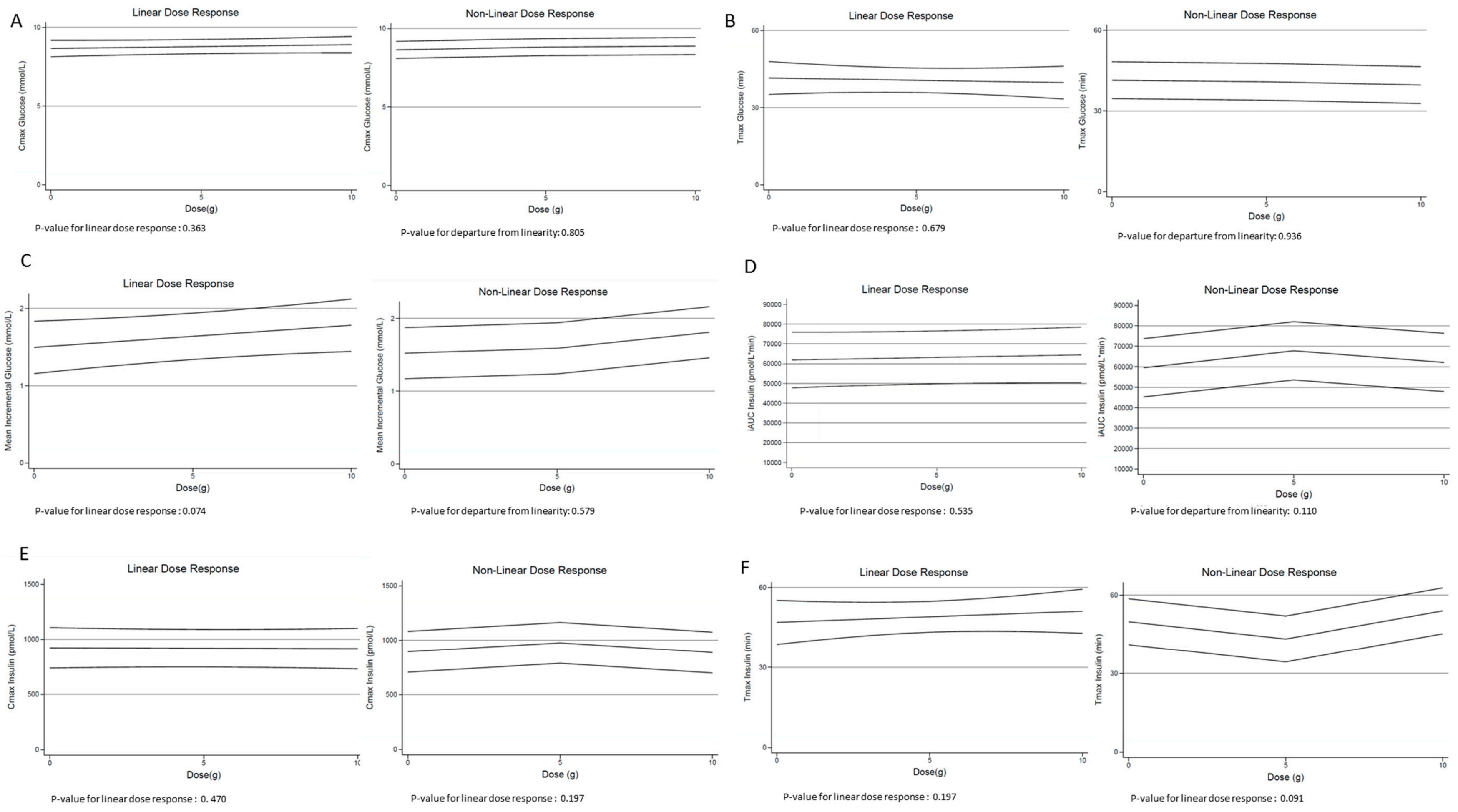

Figure S3. Linear (left) and non-linear (right) dose-response analysis of the effect of small doses of fructose on absolute C_{max} glucose (A), T_{max} glucose (B), mean incremental glucose (C), insulin iAUC (D), absolute C_{max} insulin (E) and T_{max} insulin (F). In the linear dose-response graphs, the inner line represents the predicted linear trend, while the two outer lines represent the 95% CIs. In the non-linear dose-response graphs, the center line represents the mean, while the two outer lines represent the 95% CIs. P-values correspond to log-transformed data due to non-normal distribution of residuals for (D) and (E). P-values were considered significant if P<0.0125, see Section 2.8.

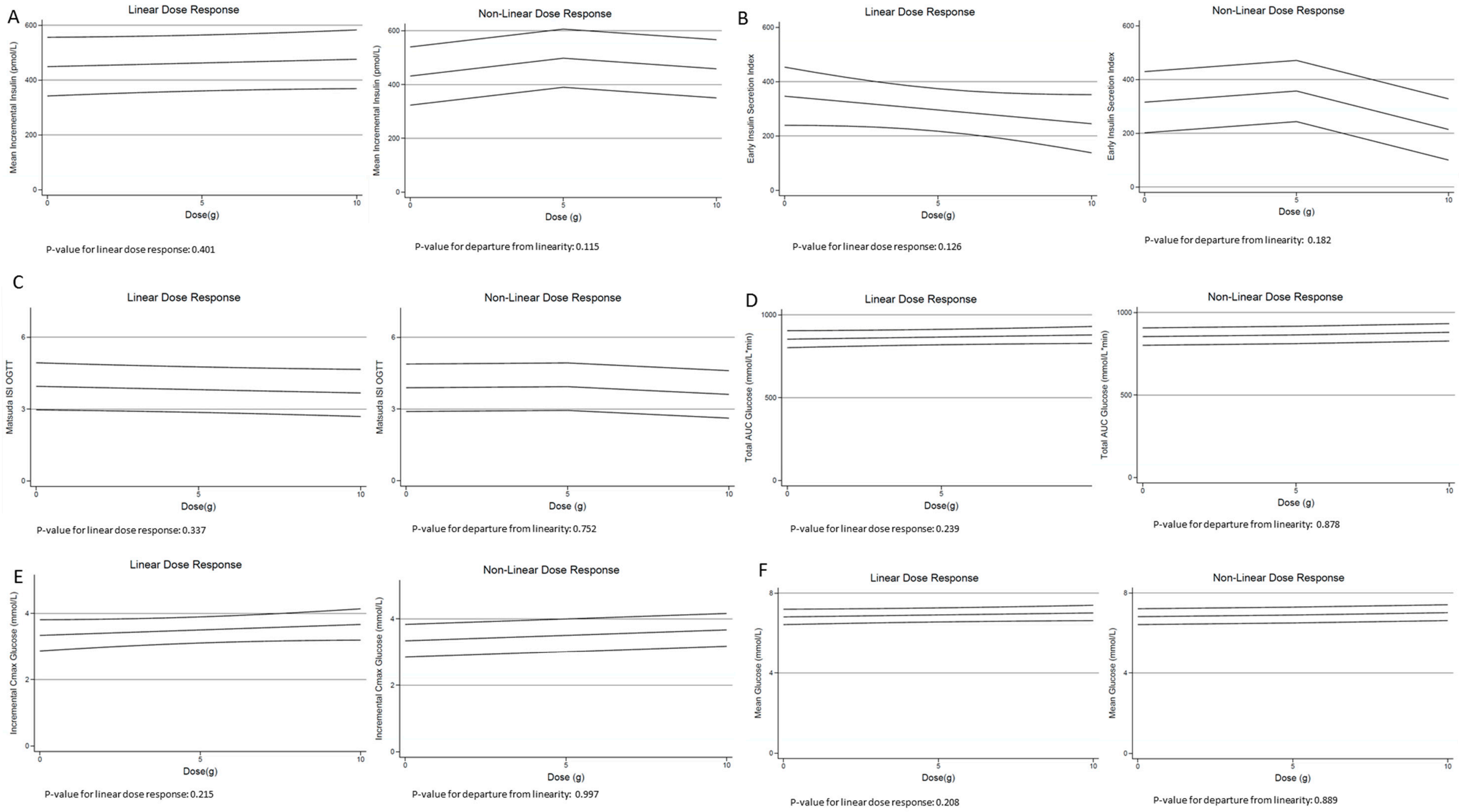

Figure S4. Linear (left) and non-linear (right) dose-response analysis of the effect of small doses of fructose mean incremental insulin (A), Early Insulin Secretion Index ($\Delta PI_{30-0}/\Delta PG_{30-0}$) (B), Matsuda Insulin Sensitivity Index$_{OGTT}$ (C), total AUC glucose (D), incremental C_{max} glucose (E) and absolute mean glucose (F). In the linear dose-response graphs, the inner line represents the predicted linear trend, while the two outer lines represent the 95% CIs. In the non-linear dose-response graphs, the center line represents the mean, while the two outer lines represent the 95% CIs. P-values correspond to log-transformed data due to non-normal distribution of residuals for (A), (B), and (C). P-values were considered significant if P<0.0125, see Section 2.8.

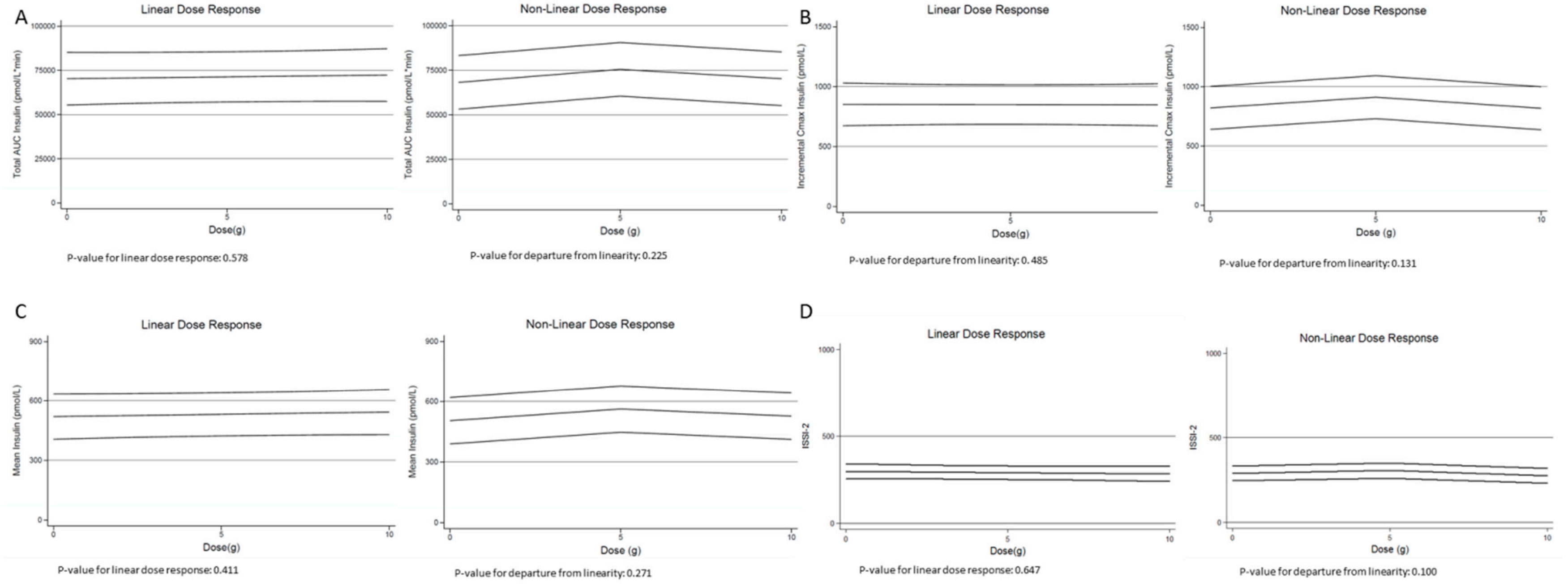

Figure S5. Linear (left) and non-linear (right) dose-response analysis of the effect of small doses of fructose on total AUC insulin (A), incremental C_{max} insulin (B), absolute mean insulin (C) and Insulin Secretion-Sensitivity Index-2 (ISSI-2) (D). In the linear dose-response graphs, the inner line represents the predicted linear trend, while the two outer lines represent the 95% CIs. In the non-linear dose-response graphs, the center line represents the mean, while the two outer lines represent the 95% CIs. P-values correspond to log-transformed data due to non-normal distribution of residuals for (A), (B), (C), and (D). P-values were considered significant if P<0.0125, see Section 2.8.

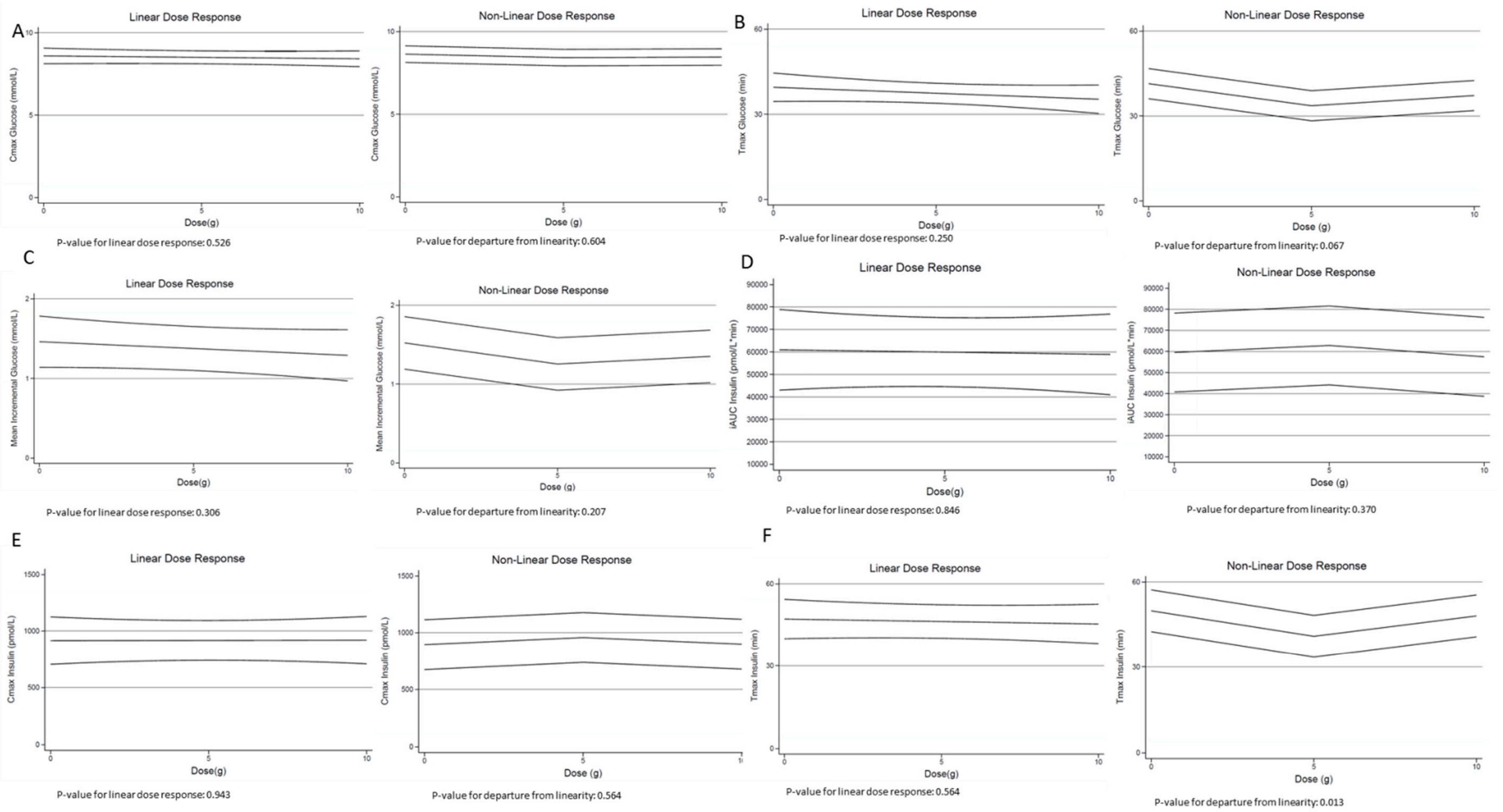

Figure S6. Linear (left) and non-linear (right) dose-response analysis of the effect of small doses of allulose on absolute C_{max} glucose (A), T_{max} glucose (B), mean incremental glucose (C), insulin iAUC (D), absolute C_{max} insulin (E) and T_{max} insulin (F). In the linear dose-response graphs, the middle black line represents the predicted linear trend, while the two outer lines represent the 95% CIs. In the non-linear dose-response graphs, the middle black line represents the mean, while the two grey lines represent the 95% CIs. P-values correspond to log-transformed data due to non-normal distribution of residuals for (D) and (E). P-values were considered significant if $P<0.0125$, see Section 2.8.

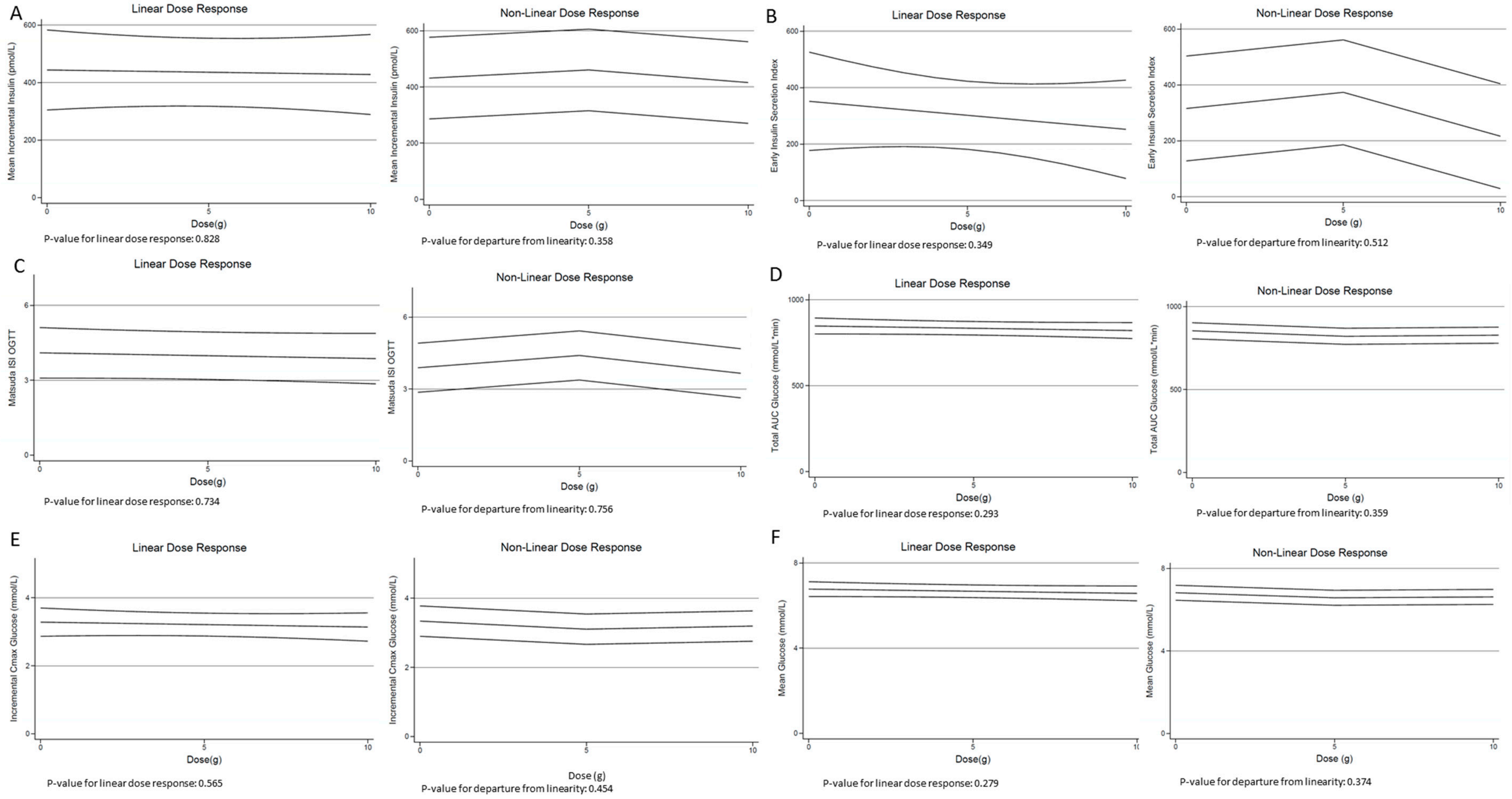

Figure S7. Linear (left) and non-linear (right) dose-response analysis of the effect of small doses of allulose on mean incremental insulin (A), Early Insulin Secretion Index ($\Delta PI_{30\text{-}0}/\Delta PG_{30\text{-}0}$) (B), Matsuda Insulin Sensitivity $Index_{OGTT}$ (C), total AUC glucose (D), incremental C_{max} glucose (E) and absolute mean glucose (F). In the linear dose-response graphs, the middle black line represents the predicted linear trend, while the two grey lines represent the 95% CIs. In the non-linear dose-response graphs, the middle black line represents the mean, while the two outer lines represent the 95% CIs. P-values correspond to log-transformed data due to non-normal distribution of residuals for (A), (B), and (C). P-values were considered significant if P<0.0125, see Section 2.8.

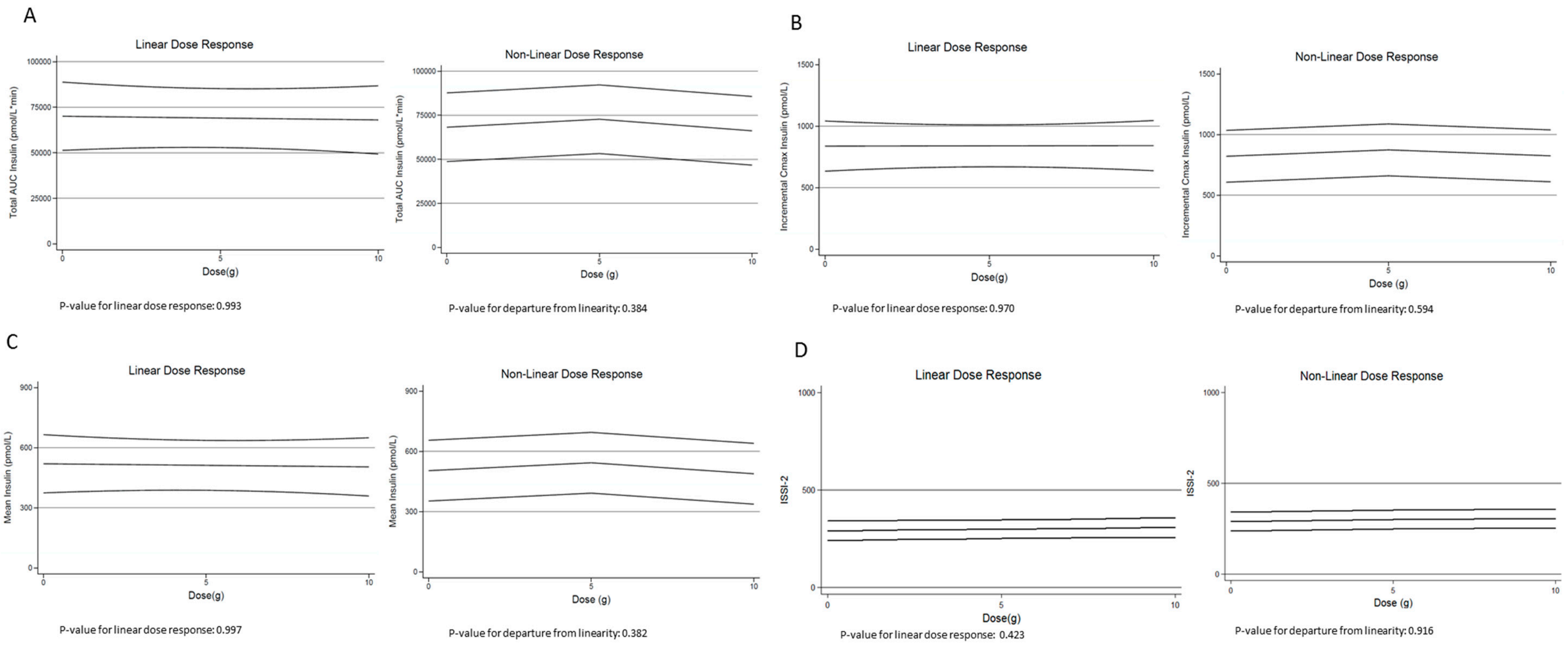

Figure S8. Linear (left) and non-linear (right) dose-response analysis of the effect of small doses of allulose on total AUC insulin (A), incremental C_{max} insulin (B), absolute mean insulin (C) and Insulin Secretion-Sensitivity Index-2 (ISSI-2) (D). In the linear dose-response graphs, the middle black line represents the predicted linear trend, while the two grey lines represent the 95% CIs. In the non-linear dose-response graphs, the middle black line represents the mean, while the two outer lines represent the 95% CIs. P-values correspond to log-transformed data due to non-normal distribution of residuals for (A), (B),(C) and (D). P-values were considered significant if P<0.0125, see Section 2.8.

Subgroup	Level	N	Mean [95% CI]	P-value
**Total**		25	20.6 [-10.0, 51.2]	
**Age, years**	< 30	12	39.8 [-3.1, 82.7]	0.22
	≥ 30	13	2.9 [-38.3, 44.1]	
**Sex**	Male	13	27.1 [-15.1, 69.4]	0.66
	Female	12	13.5 [-30.5, 57.5]	
**Ethnicity***	Caucasian	12	9.1 [-27.3, 45.6]	0.04**
	South Asian	5	15.7 [-40.8, 72.2]	
	Other	3	113.5 [40.6, 186.5]	
	East Asian	2	-61.5 [-150.8, 27.8]	
	African -American	2	69.6 [-19.8 , 158.9]	
	Caribbean	1	-30.0 [-156.3, 96.3]	
**BMI*, kg/m^2**	Normal	12	11.9 [-31.8, 55.6]	0.77
	Overweight	9	21.7 [-28.7, 72.2]	
	Obese	4	44.1 [-31.6, 119.8]	
**Baseline HbA_{1c}, %**	≤ 5.1	12	14.7 [-29.4, 58.7]	0.71
	≥ 5.1	13	26.1 [-16.3, 68.4]	
**Baseline FPG mmol/L**	< 5.5	12	29.9 [-14.0, 73.7]	0.57
	≥ 5.5	13	12.1 [-30.1, 54.2]	
**Baseline FPI pmol/L**	≤ 61.5	12	16.3 [-27.8, 60.4]	0.79
	> 61.5	13	24.6 [-17.8, 70.0]	
**Baseline HOMA-IR**	< 2.67	12	16.3 [-27.8, 60.4]	0.79
	≥ 2.67	13	24.6 [-17.8, 70.0]	

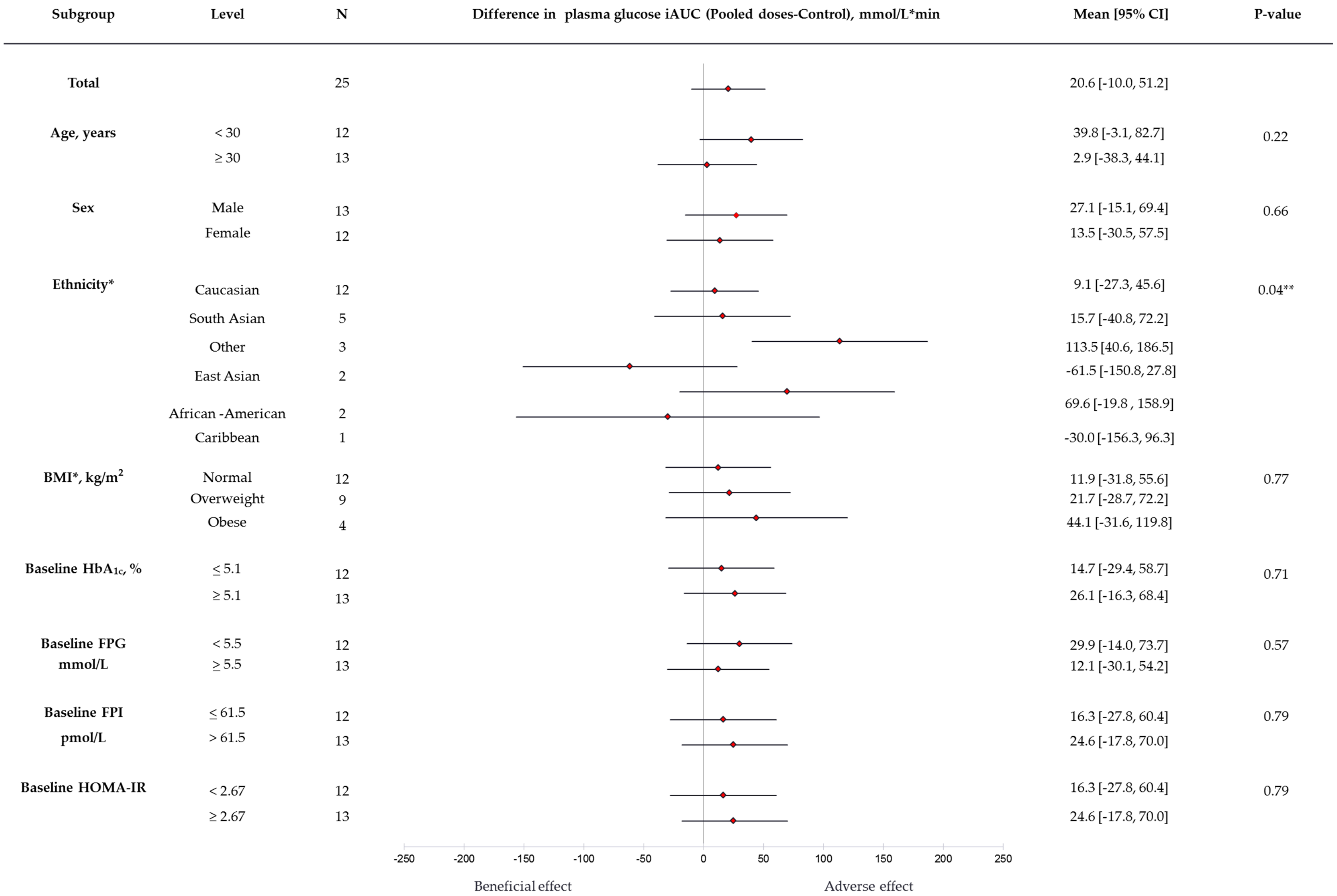

Figure S9. Post-hoc subgroup analyses for the effect of fructose on the difference in glucose iAUC between pooled doses and control. Data for HbA1c, fasting plasma glucose and insulin, and HOMA-IR are based on visit 1 data (baseline) for all subjects. The category 'Other' under ethnicity includes; Middle Eastern, South-American, and South-American/African-American. *For those from Asian descent, ethnic specific BMI cut-offs were used. Where BMI <22.9 kg/m^2 = normal, BMI 23-24.9kg/m^2 =overweight, and BMI >25kg/m^2=obese. For all other ethnicities, BMI< 24.9kg/m^2= normal, BMI ≥ 25-29.9 kg/m^2 =overweight, and BMI >30kg/m^2 =obese BMI, Body mass index; FPI, fasting plasma insulin; FPG, fasting plasma glucose; HbA1c, Glycated hemoglobin; HOMA-IR, homeostasis model assessment-insulin resistance. **P-values <0.05 were considered significant.

Subgroup	Level	N	Difference in plasma glucose iAUC (Pooled doses-Control), mmol/L*min	MD [95% CI]	P-value
**Total**		25		-28.7 [-59.0, 1.7]	
**Age, years**	< 30	12		-31.4 [-75.2, 12.4]	0.86
	≥ 30	13		-26.1 [-68.2, 16.0]	
**Sex**	Male	13		-28.6 [-72.5, 15.2]	0.99
	Female	12		-28.7 [-70.8, 13.4]	
**Ethnicity***					
	Caucasian	12		-35.1 [-74.5, 4.3]	0.31
	South Asian	5		-10.2 [-71.3, 50.8]	
	Other	3		36.6 [-42.2, 115.3]	
	East Asian	2		-31.4 [-127.9, 65.1]	
	African -American	2		-97.8 [-194.3, -1.3]	
	Caribbean	1		-95.3 [-231.7, 41.2]	
**BMI*, kg/m^2**	Normal	12		-25.4 [-68.9, 18.1]	0.84
	Overweight	9		-39.7 [-89.9, 10.6]	
	Obese	4		-13.6 [-89.1, 61.8]	
**Baseline HbA$_{1c}$, %**	≤ 5.1	12		-15.1 [-58.3, 28.1]	0.39
	≥ 5.1	13		-41.2 [-82.7, 0.4]	
**Baseline FPG mmol/L**	< 5.5	12		-25.6 [-69.4, 18.2]	0.85
	≥ 5.5	13		-31.5 [-73.6, 10.6]	
**Baseline FPI pmol/L**	≤ 61.5	12		-37.4 [-90.0, 6.2]	0.59
	> 61.5	13		-20.6 [-62.5, 21.3]	
**Baseline HOMA-IR**	< 2.67	12		-37.4 [-90.0, 6.2]	0.59
	≥ 2.67	13		-20.6 [-62.5, 21.3]	

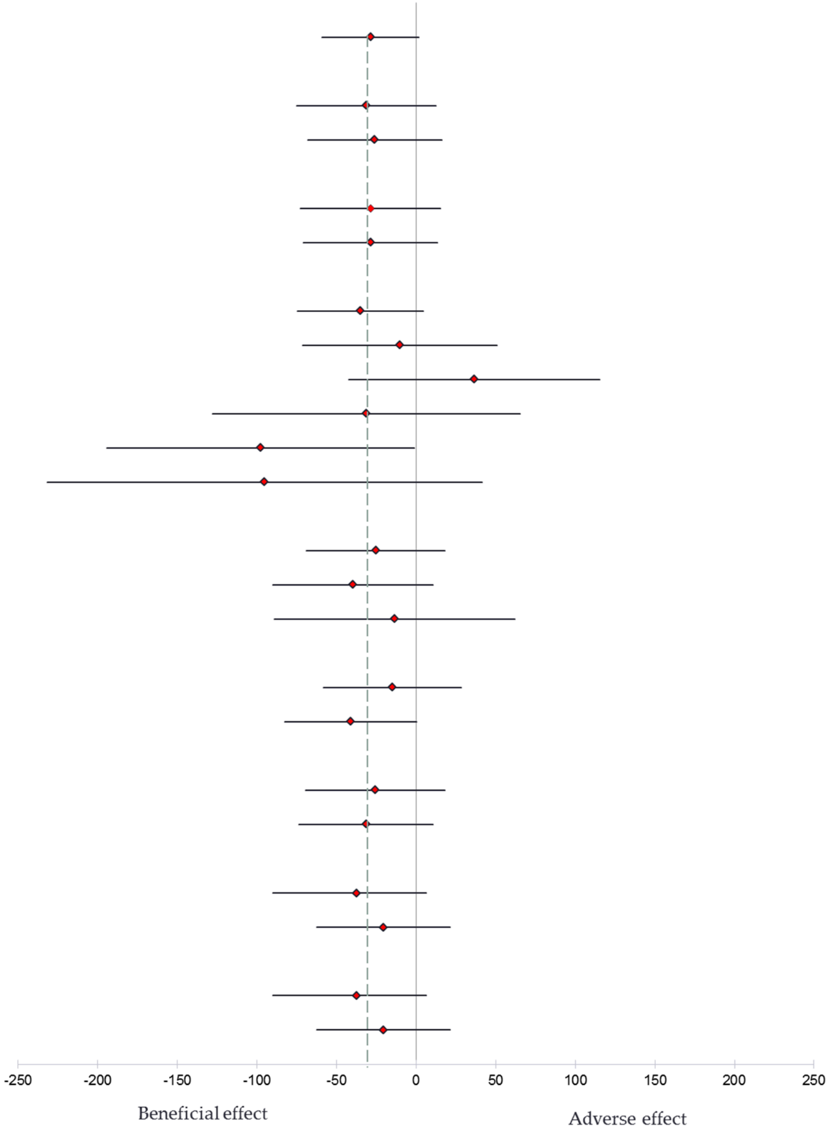

Figure S10. Post-hoc subgroup analyses for the effect of allulose on the difference in glucose iAUC between pooled doses and control. Data for HbA1c, fasting plasma glucose and insulin, and HOMA-IR are based on visit 1 data (baseline) for all subjects. The category 'Other' under ethnicity includes; Middle Eastern, South-American, and South-American/African-American. *For those of Asian descent, ethnic specific BMI cut-offs were used. Where BMI <22.9 kg/m^2 = normal, BMI 23-24.9kg/m^2 =overweight, and BMI >25kg/m^2=obese. For all other ethnicities, BMI< 24.9kg/m^2= normal, BMI ≥ 25-29.9 kg/m^2 =overweight, and BMI >30kg/m^2 =obese. BMI, Body mass index; FPI, fasting plasma insulin; FPG, fasting plasma glucose; HbA1c, Glycated hemoglobin; HOMA-IR, homeostasis model assessment-insulin resistance. P-values <0.05 were considered significant.

A. FRUCTOSE

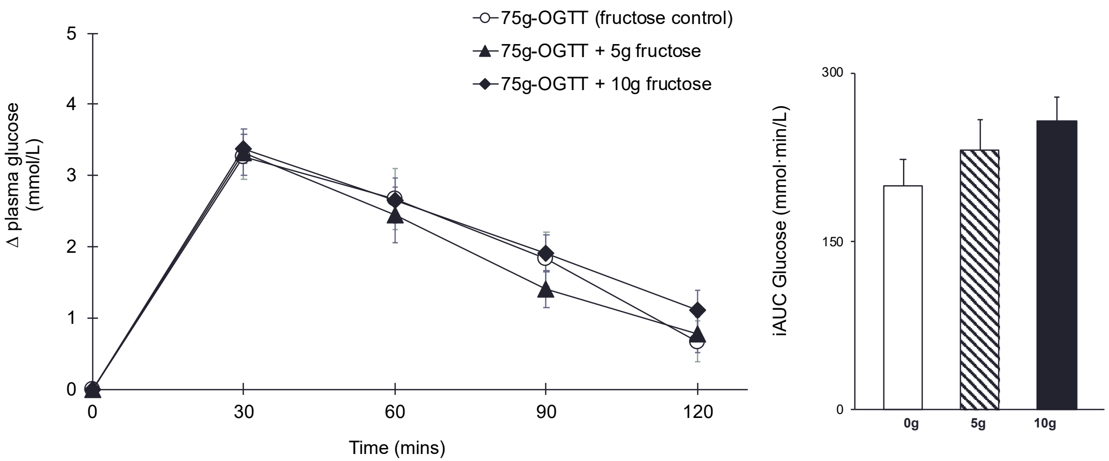

Treatment	iAUC Glucose (mmol/L*min)
75g-OGTT 0g fructose (control)	200 ± 24
75g-OGTT + 5g fructose	232 ± 27
75g-OGTT + 10g fructose	258 ± 21
Pooled fructose doses	245 ± 20
0g vs. 5g, contrast (SE)	32 ± 24
0g vs. 10g, contrast (SE)	58 ± 24
0g vs. 5g, p-value	0.18
0g vs. 10g, p-value	0.01*
Pooled fructose , p-value	0.02*

B. ALLULOSE

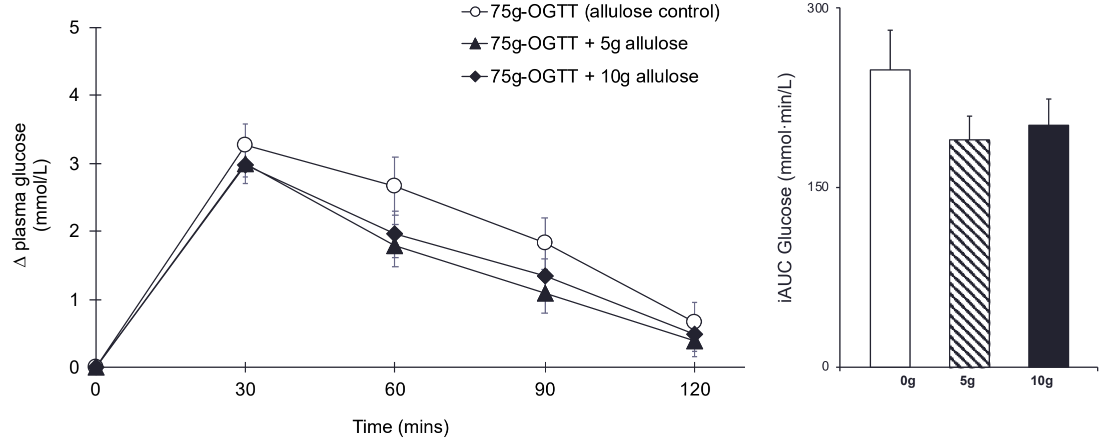

Treatment	iAUC Glucose (mmol/L*min)
75g-OGTT 0g allulose (control)	249 ± 32
75g-OGTT + 5g allulose	189 ± 20
75g-OGTT + 10g allulose	202 ± 22
Pooled allulose doses	196 ± 17
0g vs. 5g, contrast (SE)	-59 ± 28
0g vs. 10g, contrast (SE)	-47 ± 28
0g vs. 5g, p-value	0.03*
0g vs. 10g, p-value	0.09
Pooled allulose, p-value	0.05

Figure S11. A) Effect of small doses (5g and 10g) of fructose on incremental change for plasma glucose and the primary outcome of incremental area under the curve (iAUC) for plasma glucose following consumption of 75g-OGTT (assigned 0g fructose control), 75g-OGTT + 5g fructose and 75g-OGTT + 10g fructose in 25 healthy participants. B) Effect of small doses (5g and 10g) of allulose on incremental change in plasma glucose and the primary outcome of incremental area under the curve (iAUC) for plasma glucose following consumption of 75g-OGTT (assigned 0g allulose control), 75g-OGTT + 5g allulose and 75g-OGTT + 10g allulose in 25 healthy participants. Data reported as mean ± SEM. *P-values <0.05 are considered significant, see Section 2.8. Note that 5-10g of fructose is 1-2% of total daily calories and allulose given at doses of 5-10g contributes 0.05-0.1% of total daily calories in a 2000kcal diet.

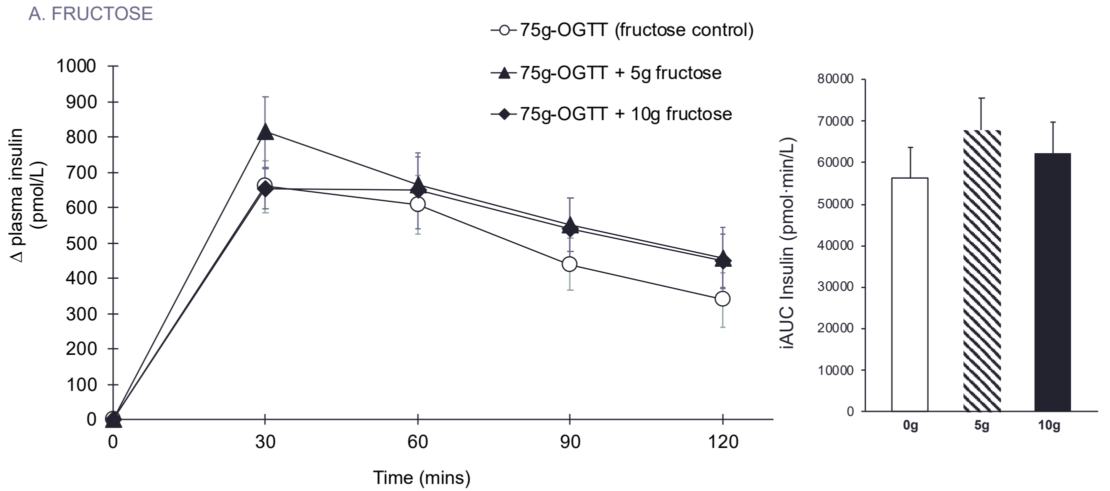

Treatment	iAUC Insulin (pmol/L*min)
75g-OGTT 0g fructose (control)	56299 ± 7336
75g-OGTT + 5g fructose	67795 ± 7825
75g-OGTT + 10g fructose	62097 ± 7644
Pooled fructose doses	64946 ± 7480
0g vs. 5g, contrast (SE)	11496 ± 4877
0g vs. 10g, contrast (SE)	5798 ± 4877
0g vs. 5g, p-value	0.01*
0g vs. 10g, p-value	0.08
Pooled fructose, p-value	0.05

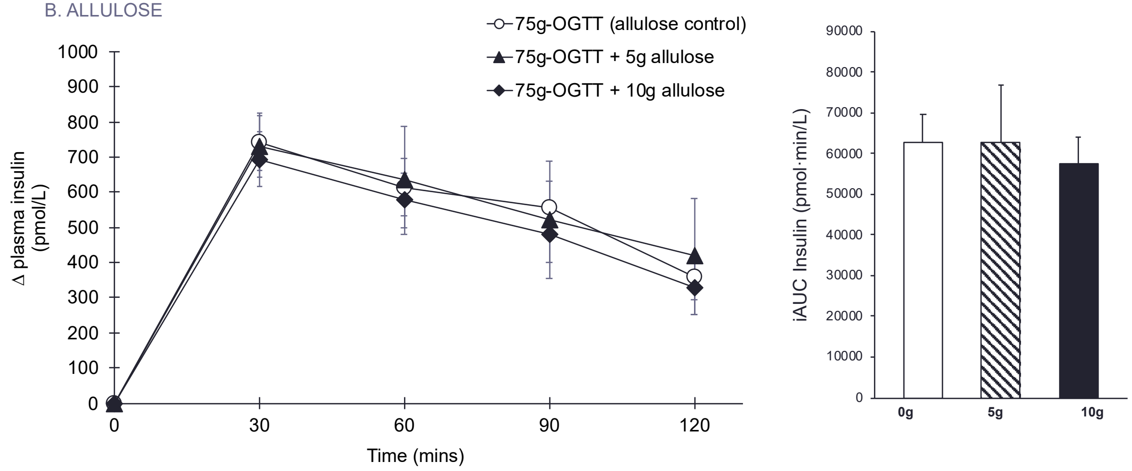

Treatment	iAUC Insulin (pmol/L*min)
75g-OGTT 0g allulose (control)	62696 ± 6853
75g-OGTT + 5g allulose	62844 ± 14042
75g-OGTT + 10g allulose	57451 ± 6560
Pooled allulose doses	60147 ± 9050
0g vs. 5g, contrast (SE)	147 ± 9652
0g vs. 10g, contrast (SE)	-5246 ± 9652
0g vs. 5g, p-value	0.19
0g vs. 10g, p-value	0.50
Pooled allulose, p-value	0.42

Figure S12. A) Effect of small doses (5g and 10g) of fructose on the secondary outcomes of incremental change and incremental area under the curve (iAUC) for plasma insulin following consumption of 75g-OGTT (assigned 0g fructose control), 75g-OGTT + 5g fructose and 75g-OGTT + 10g fructose in 25 healthy participants. **B)** Effect of small doses (5g and 10g) of allulose on the secondary outcomes of incremental change and incremental area under the curve (iAUC) for plasma insulin following consumption of 75g-OGTT (assigned 0g allulose control), 75g-OGTT + 5g allulose and 75g-OGTT + 10g allulose in 25 healthy participants. Data reported as mean ± SEM. Note that all p-values are from log-transformed data due to non-normality of residuals. P-values <0.0125 are considered significant, see Section 2.8. *P-value was rounded down from p=0.014 and is not considered significant. Note that 5-10g of fructose is 1-2% of total daily calories and allulose given at doses of 5-10g contributes 0.05-0.1% of total daily calories in a 2000kcal diet.

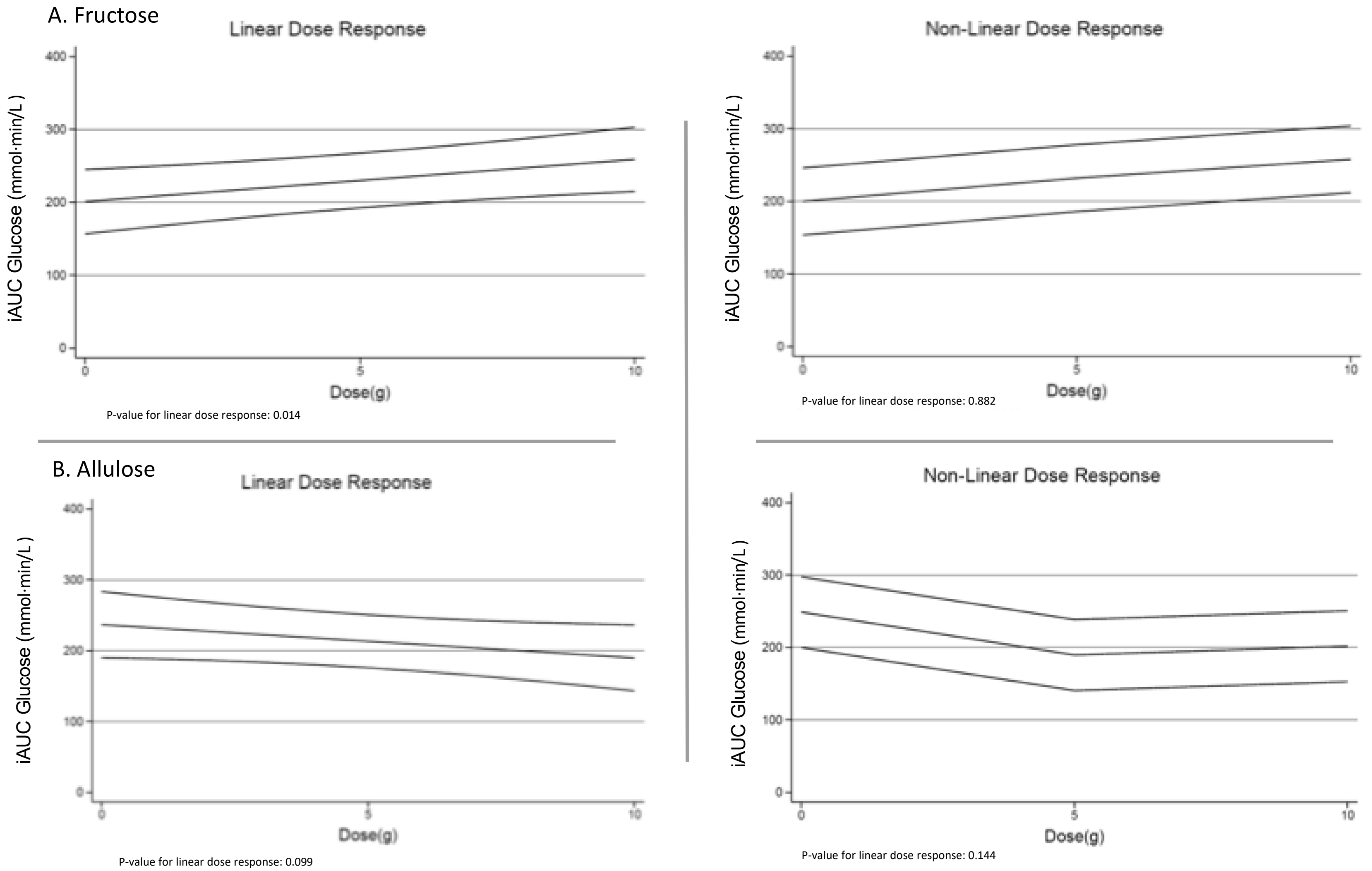

Figure S13. A) Linear (left) and non-linear dose-response (right) analysis of the effect of small doses of fructose on plasma glucose iAUC in a sensitivity analysis using the assigned fructose control. B) Linear (left) and non-linear (right) dose-response analysis of the effect of small doses of allulose on plasma glucose iAUC in a sensitivity analysis using the assigned allulose control. In the linear dose-response graphs, the middle black line represents the predicted linear trend, while the two grey lines represent the 95% CIs. In the non-linear dose-response graphs, the middle black line represents the mean, while the two grey lines represent the 95% CIs. P-values were considered significant if $P<0.0125$, see Section 2.8.

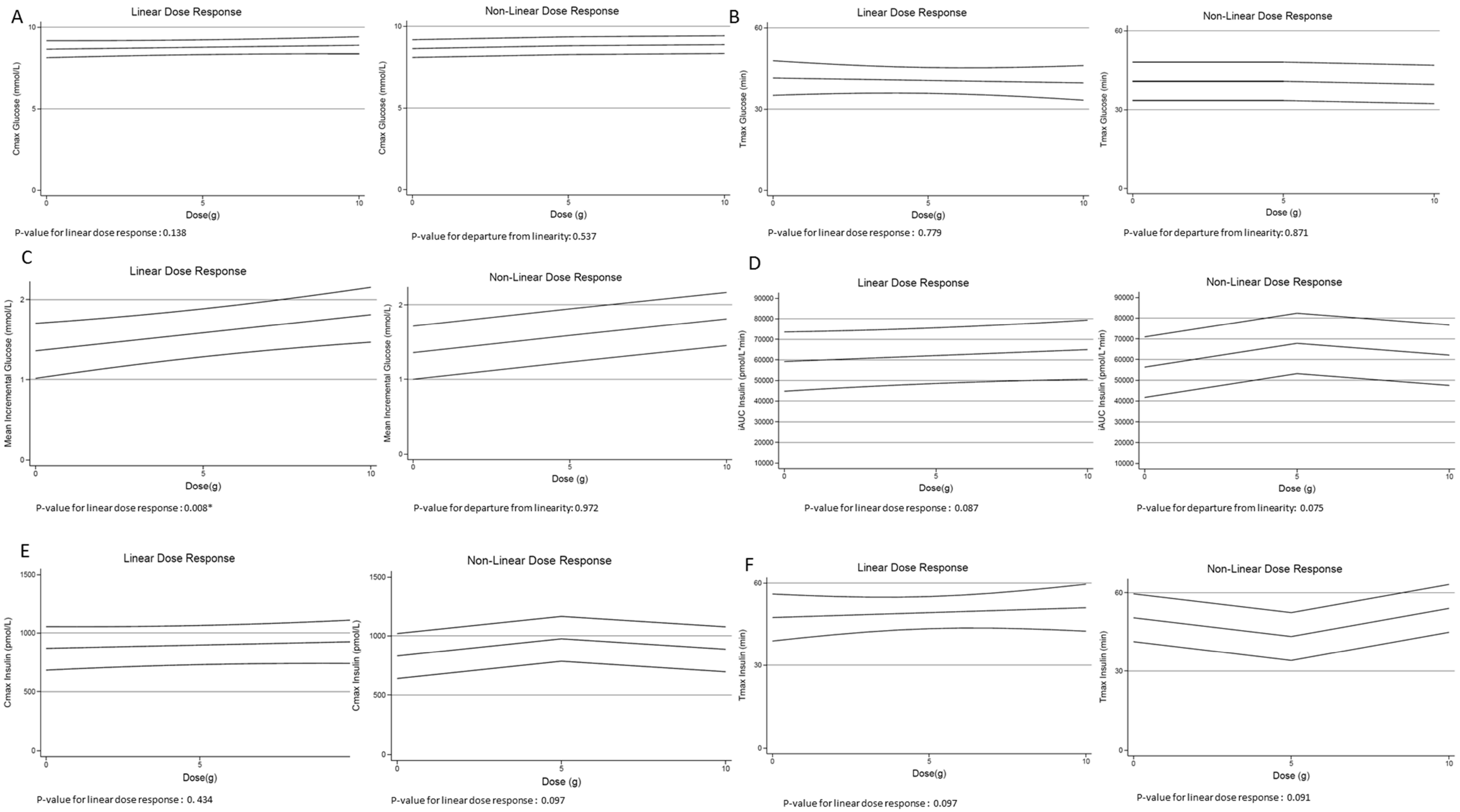

Figure S14. Linear (left) and non-linear (right) dose-response analysis of the effect of small doses of fructose on absolute C_{max} glucose (A), T_{max} glucose (B), mean incremental glucose (C), insulin iAUC (D), absolute C_{max} insulin (E) and T_{max} insulin (F). This sensitivity analysis used the assigned fructose 0g-control in the dose response analysis. In the linear dose-response graphs, the middle black line represents the predicted linear trend, while the two outer lines represent the 95% CIs. In the non-linear dose-response graphs, the middle black line represents the mean, while the two outer lines represent the 95% CIs. P-values correspond to log-transformed data due to non-normal distribution of residuals for (D) and (E). *P-values <0.05 are considered significant, see Section 2.8.

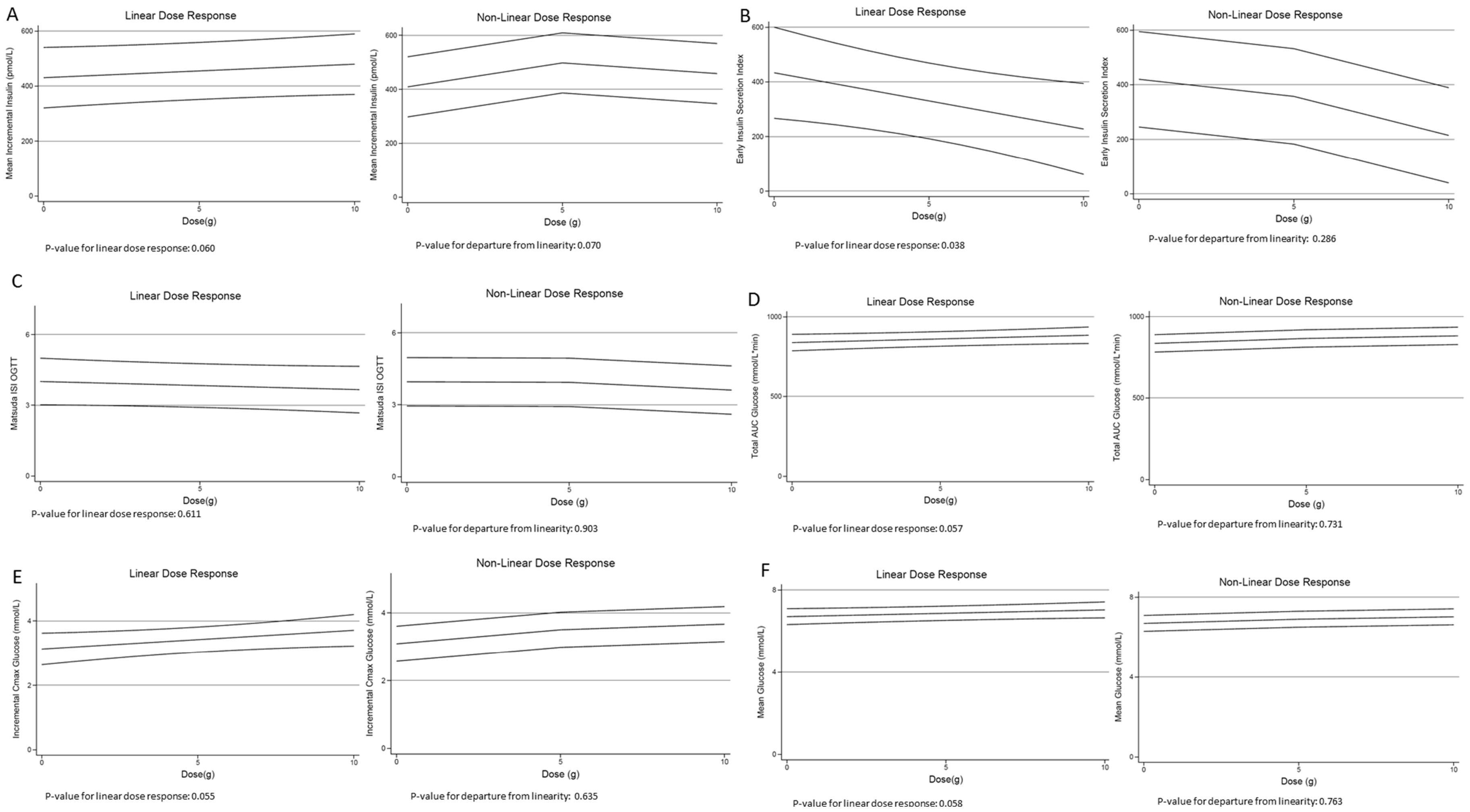

Figure S15. Linear (left) and non-linear (right) dose-response analysis of the effect of small doses of fructose mean incremental insulin (A), Early Insulin Secretion Index ($\Delta PI_{30-0}/\Delta PG_{30-0}$) (B), Matsuda Insulin Sensitivity Index$_{OGTT}$ (C), total AUC glucose (D), incremental C_{max} glucose (E) and absolute mean glucose (F). This sensitivity analysis used the assigned fructose 0g-control in the dose response analysis. In the linear dose-response graphs, the inner line represents the predicted linear trend, while the two outer lines represent the 95% CIs. In the non-linear dose-response graphs, the center line represents the mean, while the two outer lines represent the 95% CIs. P-values correspond to log-transformed data due to non-normal distribution of residuals for (A), (B), and (C). P-values <0.05 are considered significant, see Section 2.8.

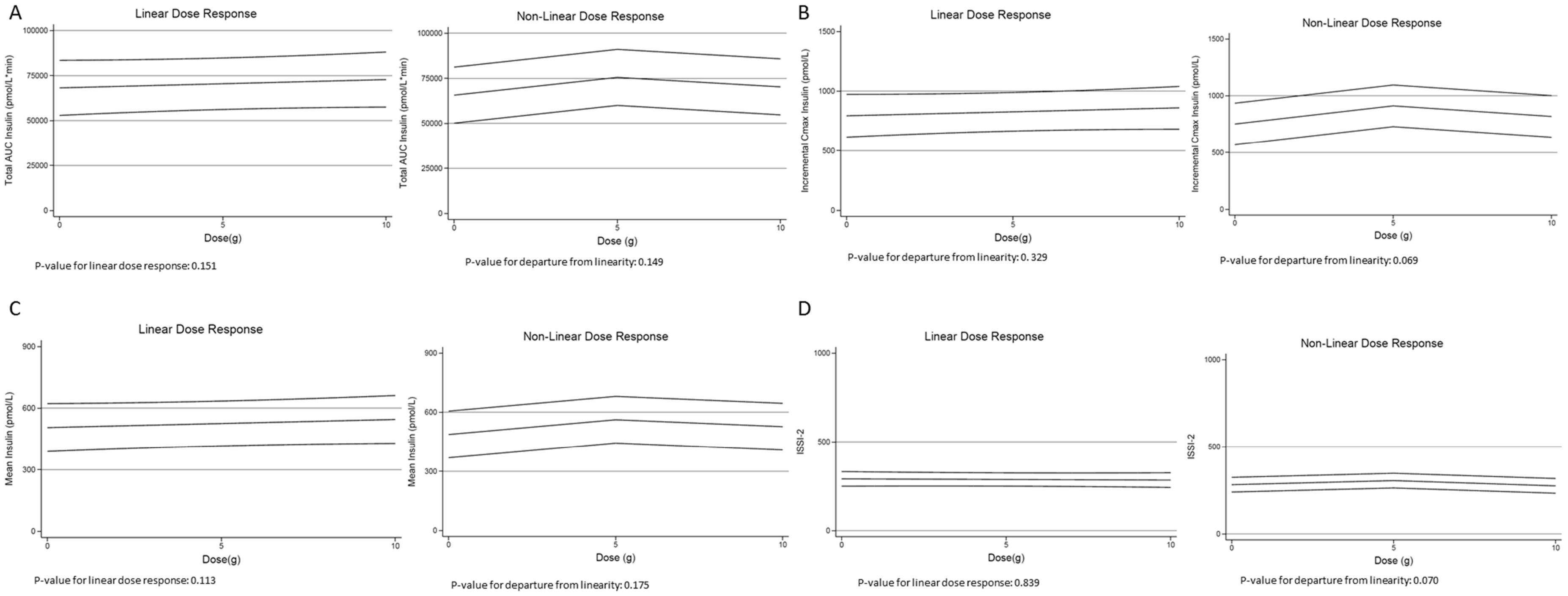

Figure S16. Linear (left) and non-linear (right) dose-response analysis of the effect of small doses of fructose on total AUC insulin (A), incremental C_{max} insulin (B), absolute mean insulin (C) and Insulin Secretion-Sensitivity Index-2 (ISSI-2) (D). This sensitivity analysis used the assigned fructose 0g-control in the dose response analysis. In the linear dose-response graphs, the inner line represents the predicted linear trend, while the two outer lines represent the 95% CIs. In the non-linear dose-response graphs, the center line represents the mean, while the two outer lines represent the 95% CIs. P-values correspond to log-transformed data due to non-normal distribution of residuals for (A), (B), (C), and (D). P-values <0.05 are considered significant, see Section 2.8.

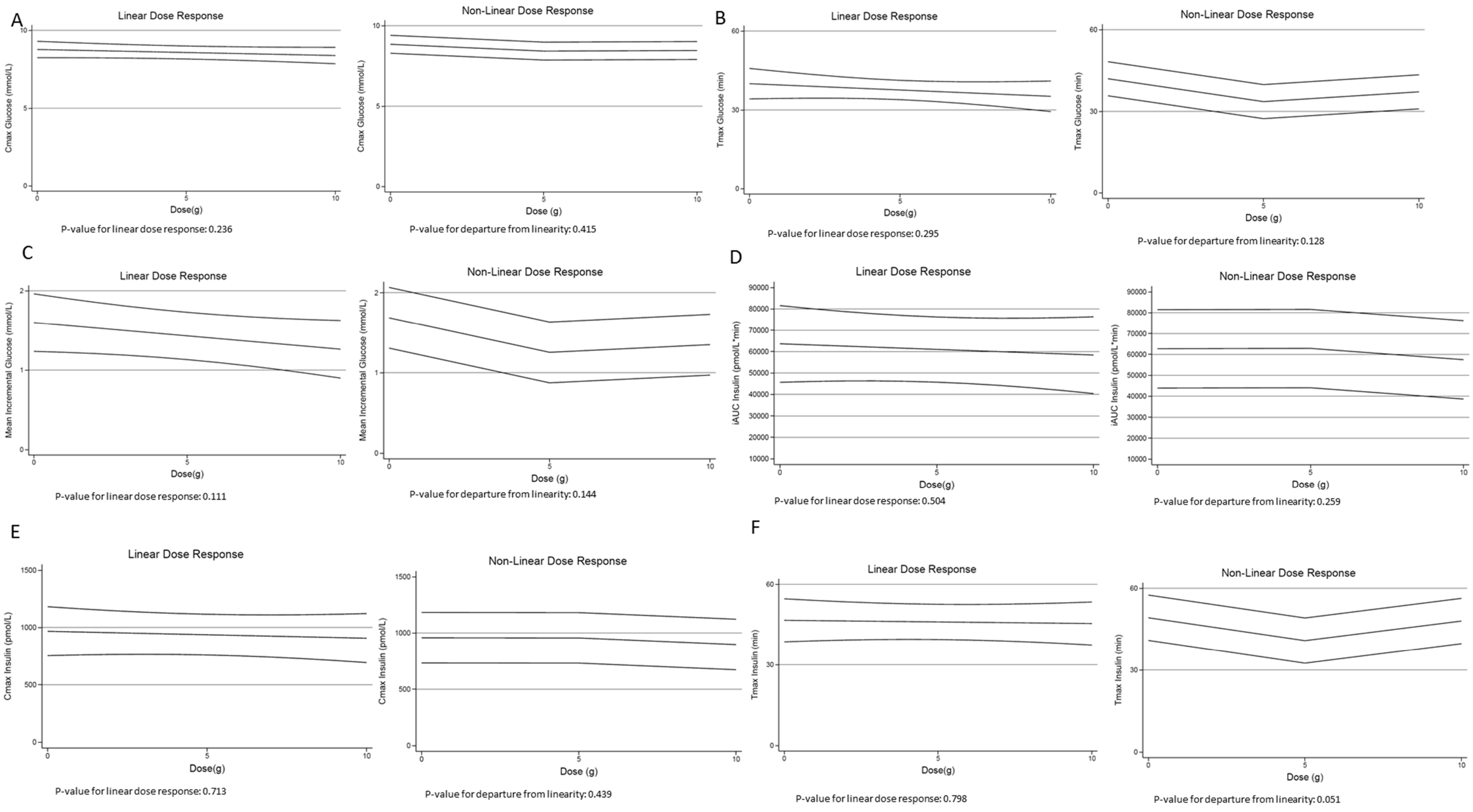

Figure S17. Linear (left) and non-linear (right) dose-response analysis of the effect of small doses of allulose on absolute Cmax glucose (A), Tmax glucose (B), mean incremental glucose (C), insulin iAUC (D), absolute Cmax insulin (E) and Tmax insulin (F). This sensitivity analysis used the assigned allulose 0g-control in the dose response analysis. In the linear dose-response graphs, the middle black line represents the predicted linear trend, while the two outer lines represent the 95% CIs. In the non-linear dose-response graphs, the middle black line represents the mean, while the two grey lines represent the 95% CIs. P-values correspond to log-transformed data due to non-normal distribution of residuals for (D) and (E). P-values <0.05 are considered significant, see Section 2.8.

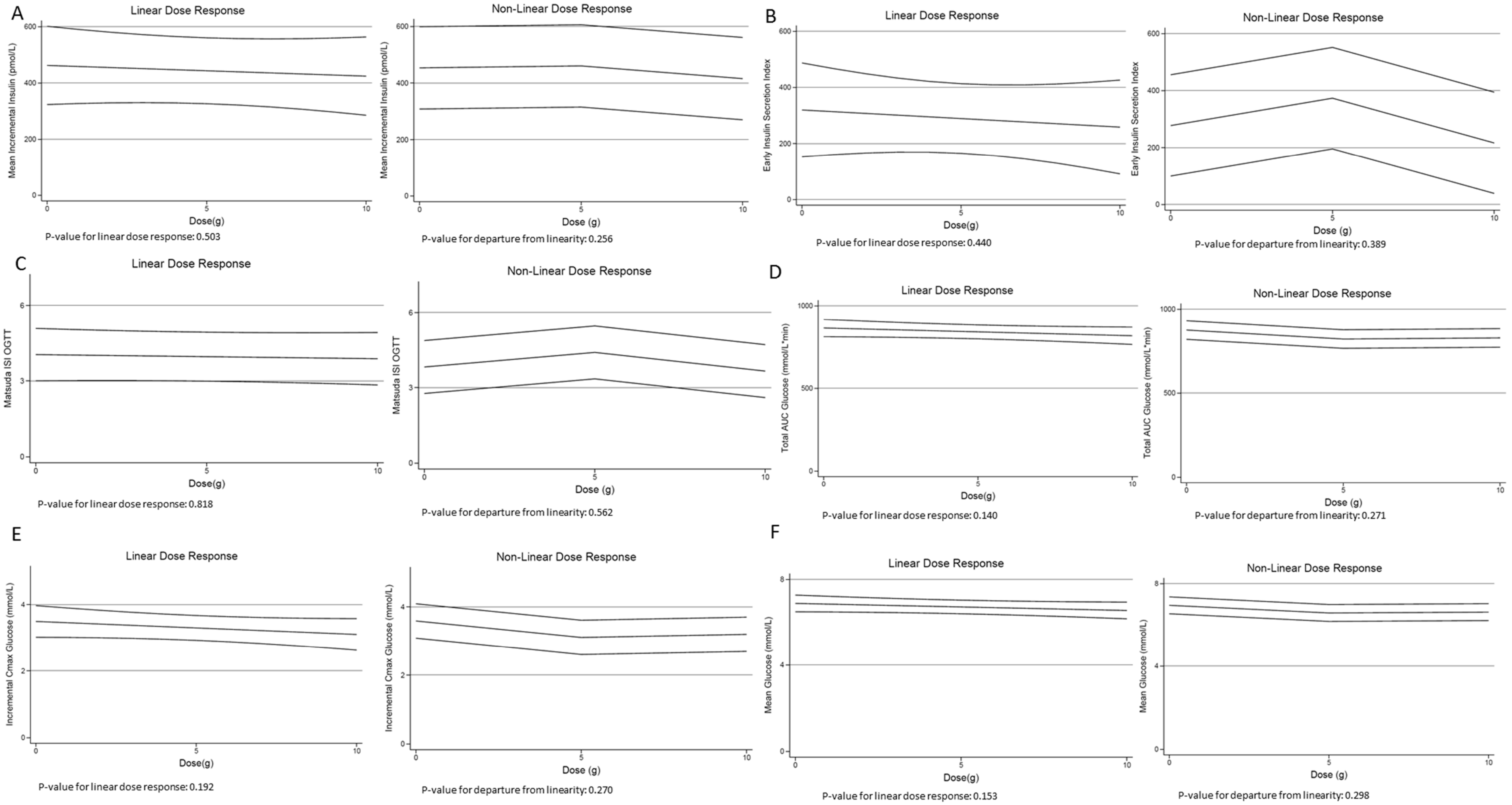

Figure S18. Linear (left) and non-linear (right) dose-response analysis of the effect of small doses of allulose on mean incremental insulin (A), Early Insulin Secretion Index ($\Delta PI_{30-0}/\Delta PG_{30-0}$) (B), Matsuda Insulin Sensitivity Index$_{OGTT}$ (C), total AUC glucose (D), incremental C_{max} glucose (E) and absolute mean glucose (F). This sensitivity analysis used the assigned allulose 0g-control in the dose response analysis. In the linear dose-response graphs, the middle black line represents the predicted linear trend, while the two grey lines represent the 95% CIs. In the non-linear dose-response graphs, the middle black line represents the mean, while the two outer lines represent the 95% CIs. P-values correspond to log-transformed data due to non-normal distribution of residuals for (A), (B), and (C). P-values <0.05 are considered significant, see Section 2.8.

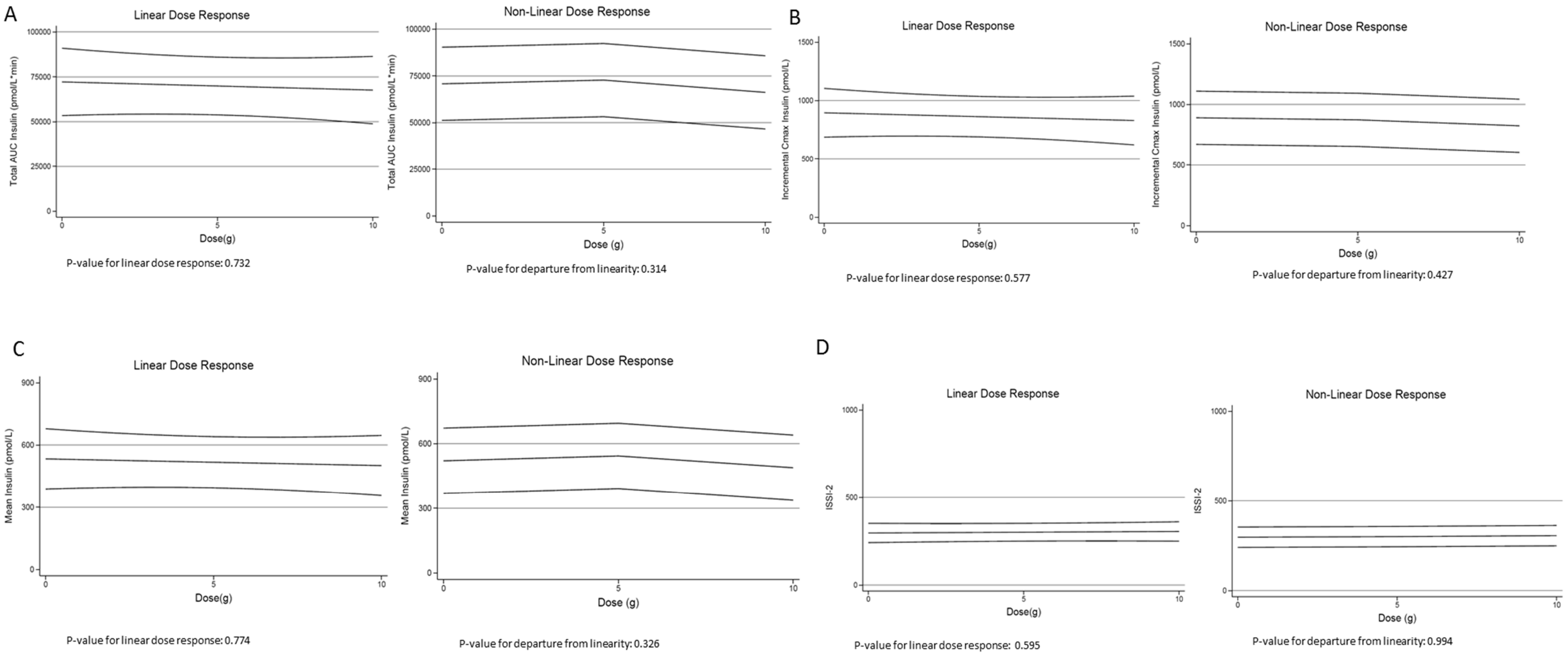

Figure S19. Linear (left) and non-linear (right) dose-response analysis of the effect of small doses of allulose on total AUC insulin (A), incremental C_{max} insulin (B), absolute mean insulin (C) and Insulin Secretion-Sensitivity Index-2 (ISSI-2) (D). This sensitivity analysis used the assigned allulose 0g-control in the dose response analysis. In the linear dose-response graphs, the middle black line represents the predicted linear trend, while the two grey lines represent the 95% CIs. In the non-linear dose-response graphs, the middle black line represents the mean, while the two outer lines represent the 95% CIs. P-values correspond to log-transformed data due to non-normal distribution of residuals for (A), (B),(C) and (D). P-values <0.05 are considered significant, see Section 2.8.

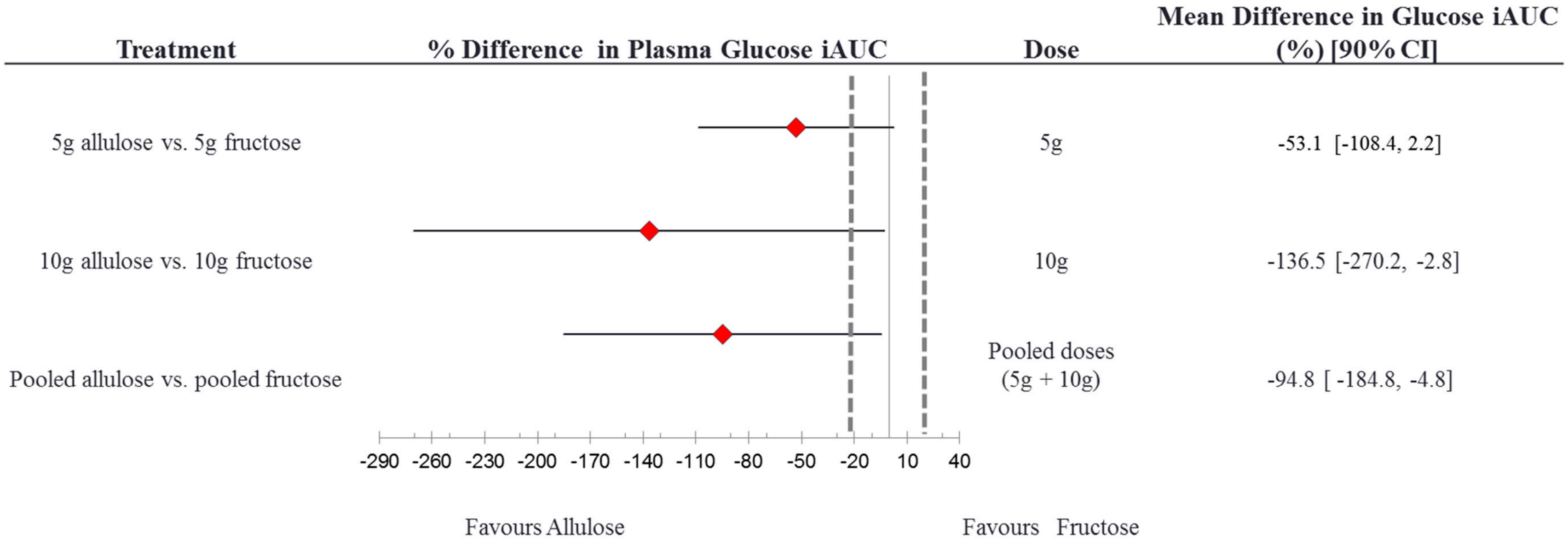

Figure S20. Equivalence test comparing the effect of allulose to fructose on plasma glucose iAUC. % difference plasma glucose iAUC = $[(\text{allulose}_{\text{iAUCglucose}}/\text{control}_{\text{iAUCglucose}}) – (\text{fructose}_{\text{iAUCglucose}}/\text{control}_{\text{iAUCglucose}})] \times 100\%$. The assigned 0g fructose and 0g allulose controls were used for this analysis. The dotted lines represent the ± 20% equivalence margins. The red diamond represents the mean difference and the black line crossing through the diamond represents the 90% CI. CI, confidence interval; iAUC, incremental area under the curve.
